# Excitatory-inhibitory balance modulates the formation and dynamics of neuronal assemblies in cortical networks

**DOI:** 10.1126/sciadv.abg8411

**Published:** 2021-11-03

**Authors:** Sadra Sadeh, Claudia Clopath

**Affiliations:** Bioengineering Department, Imperial College London, London SW7 2AZ, UK.

## Abstract

Repetitive activation of subpopulations of neurons leads to the formation of neuronal assemblies, which can guide learning and behavior. Recent technological advances have made the artificial induction of these assemblies feasible, yet how various parameters of induction can be optimized is not clear. Here, we studied this question in large-scale cortical network models with excitatory-inhibitory balance. We found that the background network in which assemblies are embedded can strongly modulate their dynamics and formation. Networks with dominant excitatory interactions enabled a fast formation of assemblies, but this was accompanied by recruitment of other non-perturbed neurons, leading to some degree of nonspecific induction. On the other hand, networks with strong excitatory-inhibitory interactions ensured that the formation of assemblies remained constrained to the perturbed neurons, but slowed down the induction. Our results suggest that these two regimes can be suitable for computational and cognitive tasks with different trade-offs between speed and specificity.

## Teaser

Behaviorally relevant network-wide plasticity can be modulated by regimes of excitatory-inhibitory balance in cortical networks.

## INTRODUCTION

Neuronal assemblies, subgroups of coactive and interconnected neurons, are building blocks of computation and learning in the brain (*1*–*4*). Recent technological advances have provided us with unprecedented tools to bidirectionally interact with their circuitry by enabling us to record from and perturb the activity of subpopulations of neurons (*5*–*7*) to ultimately link their dynamics to behavior (*8*–*10*). Specifically, these advances allow experimentalists to artificially induce neuronal assemblies by targeted activation of a subset of cortical neurons (*10*, *11*). Efficient induction of these assemblies can provide a powerful means to trigger or suppress a specific behavior (*8*, *9*) and can potentially guide us in understanding brain diseases (*12*). As with other perturbation techniques (*13*, *14*), it is crucial to understand how parameters of stimulation, including the pattern of activation of neurons and the general state of network dynamics, can be optimized for an efficient induction.

This optimization can, however, be difficult given the complex connectivity and dynamics of cortical networks. Neuronal assemblies cannot form in isolation, as they are embedded in a background of connections from other neurons in local and distal networks, which can modulate their activity. The background network itself can be in different operating regimes (e.g., awake versus anesthetized) and modulated by different mechanisms like top-down input and neuromodulation. Moreover, perturbation of a subset of neurons embedded in these background networks is likely to create a cascade of activation of downstream neurons (*15*, *16*), which can, in turn, affect the activity and plasticity of the initially perturbed neurons in a recurrent manner. This complex interaction is especially expected in cortical networks with strong recurrent excitatory and inhibitory connections, as reported in many brain regions (*16*–*20*).

**Fig. 1.**
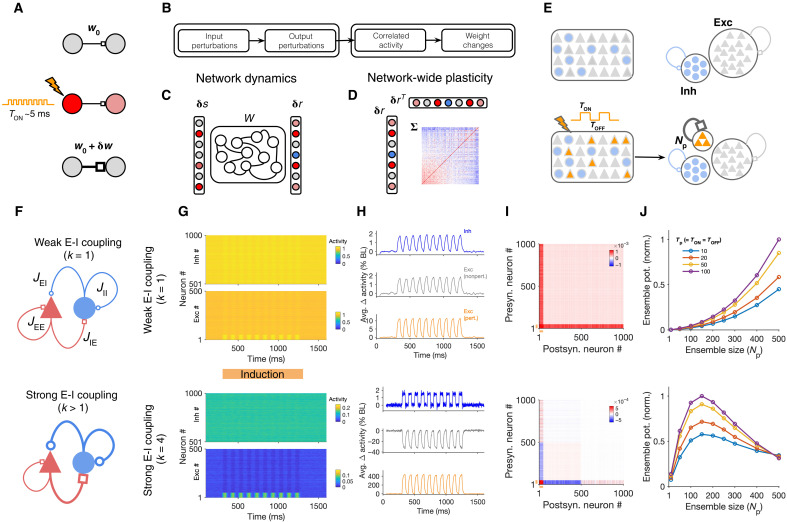
Induction of neuronal assemblies in different regimes of excitation-inhibition balance. (**A**) Schematic of conventional protocols for the induction and investigation of plasticity often involving a small number of neurons and perturbations with brief pulses. (**B** to **D**) Analytical steps (B) to evaluate the effect of external perturbations on the formation of assemblies involving dynamics of network responses (C) and network-wide plasticity (D). Knowing the weight matrix (*W*), input perturbations (δ*s*) are transferred to output perturbations (δ*r*) (C); the resulting correlated activity patterns of pre- and postsynaptic neurons (Σ), in turn, guide a network-wide plasticity of weights (D). (**E**) Schematic of the perturbation protocol to study plasticity in large-scale networks composed of excitatory (Exc) and inhibitory (Inh) neurons (top). Connections between subpopulations are not shown. Bottom left: *N*_p_ excitatory neurons are perturbed with a series of stimulation pulses alternating between ON and OFF states. Bottom right: The result of perturbations in terms of the induction of assemblies is assayed by evaluating the potentiation of weights within the perturbed ensemble of neurons (orange) as a result of Hebbian learning (see Materials and Methods). (**F**) Parameterization of different regimes in which neuronal assemblies are induced illustrating weak (*k* = 1; top) versus strong (*k* > 1; bottom) E-I coupling regimes. *J*_IE_ = ∣*J*_EI_∣ = ∣*J*_II_∣ = *kJ*_EE_. (**G**) Responses of *N*_E_ excitatory and *N*_I_ inhibitory neurons in a network with weak (*k* = 1; top) or strong (*k* = 4; bottom) E-I coupling to perturbations (10 pulses with *T*_p_ = 50, delivered to *N*_p_ = 50 neurons, starting from *T* = 300). *N*_E_ = *N*_I_ = 500. (**H**) Average normalized change in the activity (Δ activity) of different subpopulations of neurons during induction relative to the baseline (BL). (**I**) Matrix of the covariance of response changes after perturbations in (G) and (H). Orange bars, perturbed ensembles. (**J**) Assembly formation is quantified by ensemble potentiation (Ensemble pot.; see Materials and Methods, Eq. 6) for different sizes of perturbed ensembles (*N*_p_) and temporal profiles (*T*_p_).

To study the formation of neuronal assemblies under biologically realistic conditions, we therefore need to consider this complex interplay of network dynamics and plasticity. Conventional plasticity protocols, in contrast, typically study the weight changes under artificial conditions (*21*–*23*). The plasticity is often induced in a few, isolated pairs of neurons, in conditions where the effect of background activity (*24*) and network interactions is masked or minimized. They also involve patterns (*25*) and conditions (*26*) of stimulation that are optimized to maximally drive the activity of pre- and postsynaptic neurons (Fig. 1A). Learning in naturalistic conditions, on the other hand, is likely to be guided by a different pattern of neuronal perturbations (*25*), involving sparse activity of a large subpopulation of neurons (*27*), spanning a wide range of spatial and temporal scales pertinent to behaviorally relevant stimuli (*28*).

To bridge this gap, here, we studied how neuronal assemblies can be induced in large-scale recurrent networks of excitatory and inhibitory neurons under various regimes of perturbation. We used a theory that we recently developed to analyze the effect of neuronal perturbations in these networks (*29*) and asked how activity changes resulting from different perturbations guide network-wide plasticity (Fig. 1, B to D). Network response dynamics operate on much faster time scales (~10 to 100 ms) compared to the time scale of long-term plasticity (minutes to days), so we used separation of time scales between the two stages to guide our analysis. In the first step, we assessed how input perturbations are transferred to output responses, assuming a fixed weight matrix for the network (Fig. 1C); we then studied how the correlated activity patterns emerging from these responses lead to modification of neuronal ensembles (Fig. 1D). We started our study by analyzing the influence of network dynamics on network-wide plasticity (Fig. 1B) but later also considered the bidirectional interaction of the two over longer time scales.

## RESULTS

### Induction of neuronal assemblies in excitatory-inhibitory networks

We studied the formation of neuronal assemblies as a result of different patterns of perturbations in large-scale cortical network models with balance of excitation (E) and inhibition (I) (*30*, *31*). We simulated linear-rectified rate-based networks composed of E and I neurons with random recurrent connectivity (see Materials and Methods). All neurons in the network receive a background input in the baseline state before induction. During induction, a subset of excitatory neurons is targeted by repetitive external perturbations— we refer to this subset as the “perturbed ensemble” of neurons. We then assess how the perturbations change the connectivity of the perturbed ensemble to induce “neuronal assemblies”—this is defined as the increase in the strength of connectivity between perturbed neurons (Fig. 1E).

Induction protocols are characterized by the key parameters of the perturbations including the number of targeted neurons (*N*_p_, or the size of neuronal ensemble), and the temporal properties of the stimulus, which is alternating between binary states of ON (*S* = 1, for *T*_ON_) and OFF (*S* = 0, for *T*_OFF_). The background network is parameterized by the strength of E-E weights (*J*_EE_ = *J*) and the relative strength of E-I coupling (*k*). The parameter *k* describes the dominance of the overall *E* → *I* and *I* → {E, I} couplings relative to E-E weights, with *k* = 1 denoting exact balance and *k* > 1 ensuring a dominant recurrent inhibition (Fig. 1F; see Materials and Methods). E-E coupling is strong (*J* > 1), such that, in the absence of E-I interactions (*k* = 0), the network is unstable, consistent with the observation that cortical networks operate in inhibitory-stabilized regimes of activity (*19*, *32*). We simulated the response of our large-scale rate-based networks to perturbations in two regimes of recurrent E-I interaction (Fig. 1, G to J). The first regime is equipped with the minimum amount of inhibition that is necessary to stabilize the E-E subnetwork (*k* = 1); we refer to this regime as weak E-I coupling (Fig. 1G, top). The second regime has a stronger E-I coupling (*k* = 4) (Fig. 1G, bottom), which guarantees the operation of the network away from the border of instability and enables stronger lateral inhibition.

We simulated the response of the network before and after perturbations in each regime (Fig. 1, G and H). Networks in the weak E-I coupling regime had higher baseline activity compared to the networks with strong E-I coupling, which is a result of more potent recurrent inhibition in the latter regime (Fig. 1G and fig. S1A). Perturbations increased the activity of the perturbed ensemble in both regimes but had different effects on the activity of nonperturbed E neurons (Fig. 1, G and H). Nonperturbed E neurons showed, on average, positive modulation of activity during induction in networks with weak E-I coupling (*k* = 1) (Fig. 1H, top, and fig. S1A, top); this suggests that excitatory recurrent interactions are dominant in this regime. On the other hand, nonperturbed E neurons in networks with strong E-I coupling (*k* = 4) experienced a negative modulation of their activity during perturbations (Fig. 1H, bottom, and fig. S1A, bottom); this suggests that inhibitory recurrent interactions become dominant in this regime.

Notably, both effects were stronger when increasing the size of the perturbed ensemble (*N*_p_) (fig. S1, A and B): Nonperturbed E neurons in weak and strong E-I coupling regimes had, respectively, higher and lower average activity for larger *N*_p_. Moreover, similar behavior was also seen for the perturbed E subpopulation itself: The average activity of the perturbed ensemble increased by increasing *N*_p_ in the weak E-I regime, while the activity of the perturbed ensemble decreased (but remained above baseline) for larger sizes of perturbation (fig. S1, A and B). These results suggest that perturbing a subpopulation of neurons can exert a strong lateral inhibition in networks with strong E-I coupling that this effect is stronger for larger ensemble sizes and that the recurrent inhibition is also experienced within the perturbed ensemble.

We then assessed how the covariance of response changes in each regime can guide the formation of assemblies. We evaluated this by analyzing how covariance-based Hebbian-type plasticity rules change the weight matrix as a result of pre- and postsynaptic activity changes. The learning rule updates the weights on the basis of the covariance of response changes in the pre- and postsynaptic neurons (Fig. 1I) and is only invoked between E-E pairs (see Materials and Methods, Eq. 4, for details). The strength of neuronal assembly resulting from such a plasticity rule was then quantified by calculating the potentiation of synapses within the perturbed ensemble of neurons (ensemble potentiation; see Materials and Methods, Eq. 6).

Networks with weak E-I balance (*k* = 1) showed supralinear potentiation of assemblies with increasing the size of perturbed neurons (*N*_p_), and this effect was increased for longer time intervals of perturbation (*T*_p_) (Fig. 1J). Overall, this pattern is consistent with the presence of some cooperativity in the amplification of external perturbations within the perturbed ensemble (*33*), which was suggested by the enhancement of activity in this regime (fig. S1B). In the second regime with dominant E-I coupling (*k* = 4), however, we observed a different trend. The strength of assemblies grew sublinearly with *N*_p_, plateauing when around ~30% of all excitatory neurons were perturbed and dropping for larger fractions of stimulated neurons (Fig. 1J). These results indicate that, counterintuitively, increasing the number of targeted neurons by external perturbations may not always lead to formation of stronger neuronal assemblies in E-I networks.

We observed similar results for networks with higher ratio of excitatory to inhibitory neurons (fig. S1C) in networks with sparse connectivity of E-E connections (fig. S1D) and when the network connectivity was specific rather than random (fig. S1E). Our results also hold for different variants of Hebbian rule (fig. S2). We focused our analysis in the following sections on randomly connected networks with covariance-based learning rules (see Materials and Methods).

### Transition from cooperative to suppressive regimes

**Fig. 2.**
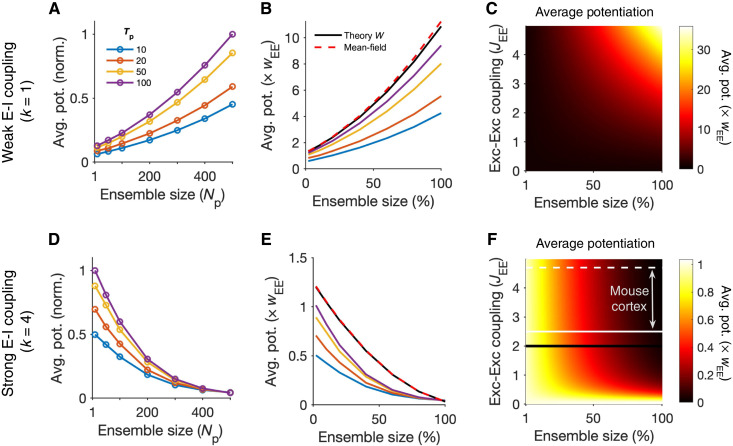
Transition from cooperative to suppressive regimes. (**A**) Average potentiation (Avg. pot.) of individual synapses within the ensemble of perturbed neurons (cf. Fig. 1J) for different ensemble sizes (*N*_p_) and temporal profiles of perturbation (*T*_p_) normalized to the maximum. (**B**) Values of average potentiation relative to the average E-E weights in the network (*w*_EE_), compared with the theoretical values obtained from linearized dynamics of the network based on its weight matrix (theory *W*) and from the mean-field analysis (dashed line) (see Materials and Methods for details). The results of simulations for larger *T*_p_ values converge to the theoretical values inferred from *W*, which, in turn, match with the mean-field analysis. Ensemble size is expressed as a fraction of total E neurons in the network (*N*_p_/*N*_E_, where *N*_E_ = 500). Other parameters are the same as Fig. 1. Networks are in the weak E-I coupling regime (*k* = 1). (**C**) Average potentiation relative to *w*_EE_ calculated from the mean-field analysis for different combination of network E-E coupling (*J*_EE_ = *N*_E_ *w*_EE_) and the size of perturbed ensembles as a fraction of the total size of the network (*N*_p_/*N*_E_). (**D** to **F**) Same as (A) to (C) for perturbed ensembles in networks with strong E-I coupling (*k* = 4). The black line in (F) corresponds to previous simulations in (D) and (E) with *J*_EE_ = 2. White lines indicate the range of *J*_EE_ estimated in mouse cortical networks with the solid and dashed lines corresponding to the mode (*J*_EE_ = 2.5) and the median (*J*_EE_ = 4.7) of the estimated values (*19*).

To gain further insights into the formation of assemblies in different regimes, we analyzed how the average strength of individual synapses changes as a function of parameters of perturbation (Fig. 2). Within the ensemble of perturbed neurons, we plotted the average potentiation of synapses for the two regimes (Fig. 2, A and D). For networks with weak E-I coupling, increasing *N*_p_ and *T*_p_ both enhanced the average potentiation (Fig. 2A), indicating that recurrent interactions amplify the strengthening of perturbed ensembles and hence the formation of assemblies. Such enhancement of potentiation per synapse combined with the increase in the number of presynaptic sources leads to the supralinear potentiation of the weights within the perturbed ensembles, as we observed before (Fig. 1J). Networks with dominant E-I coupling, on the other hand, showed a suppressive behavior per synapse: Average potentiation of synapses was decreased for larger sizes of perturbed ensembles (Fig. 2D). Combination of this suppressive effect with the increase in the number of presynaptic sources led to a sublinear growth of the total potentiation of the perturbed ensemble, as we observed before (Fig. 1J). We also observed similar dependence on the size of the perturbed ensemble for more biologically realistic implementation of networks with spiking neurons in different E/I regimes (fig. S3).

Distinct dependence of the induction on the size of the perturbed ensemble was predicted by our theoretical analysis (Fig. 2, B and E), which calculated the potentiation of synapses from the covariance of response changes resulting from the dynamics of the network given the initial weight matrix (see Materials and Methods). The results of network simulations approached the theoretical limit for larger values of *T*_p_ reflecting the fact that our analysis considers the stationary-state responses of the networks and ignores the temporal dynamics of the transients, which become more dominant in perturbations with smaller *T*_p_. Consistent with this reasoning, inferring the potentiation from very short transient responses almost abolished the dependence on the size of perturbed ensembles; conversely, very large values of *T*_p_ matched well with the theoretical prediction and numerical simulations (fig. S4). These results suggest that perturbation protocols using very fast alternating pulses may fail to reveal the effect of network dynamics on plasticity, as recurrent E/I interactions may not emerge at these short time scales.

To further understand the behavior of networks in different regimes, we developed a mean-field analysis based on the average behavior of the perturbed and nonperturbed subpopulations (see Materials and Methods). The result of the mean-field analysis matched well with the previous theoretical analysis inferred from the detailed weight matrix of the network (Fig. 2, B and E). Using the mean-field analysis, we could scan a large parameter space of arbitrarily large-scale networks with different E-E coupling and different fractions of perturbed neurons (Fig. 2, C and F). The results suggested that, for weak E-I coupling, increasing both parameters increases the average potentiation of synapses (Fig. 2C), consistent with the conventional assumption that stronger excitatory coupling and larger perturbed ensembles both enhance the formation of assemblies.

For strong E-I coupling regimes, on the other hand, we observed the opposite dependence on *N*_p_ (namely, suppression of the average potentiation for larger sizes of perturbed ensembles) for most parts of the parameter space (with the exception of a small region with very weak E-E coupling) (Fig. 2F). This relationship became steeper for stronger E-E couplings and notably held for the range of E-E couplings recently estimated in the mouse cortical networks (*19*). Combination of optogenetic perturbations with model-based inference suggested that the mean-field E-E coupling (*J*_EE_, or the self-amplification of the E population) is more than 1 (estimated median and mode of 4.7 and 2.5, respectively) (*19*), where *J*_EE_ > 1 indicates an unstable excitatory subnetwork. It is therefore possible that these networks with strong E-E connectivity show an unintuitive dependence on the size of perturbed ensembles, if they are operating in strong E-I coupling regimes. Evidence for the presence of the latter regime is indeed suggested by the functional connectivity of the mouse primary visual cortex, where perturbations of single excitatory neurons has, on average, a negative influence on other excitatory neurons (*16*), an effect that can be explained by strong E-I interactions (*29*).

### Strength versus specificity of induced ensembles

**Fig. 3.**
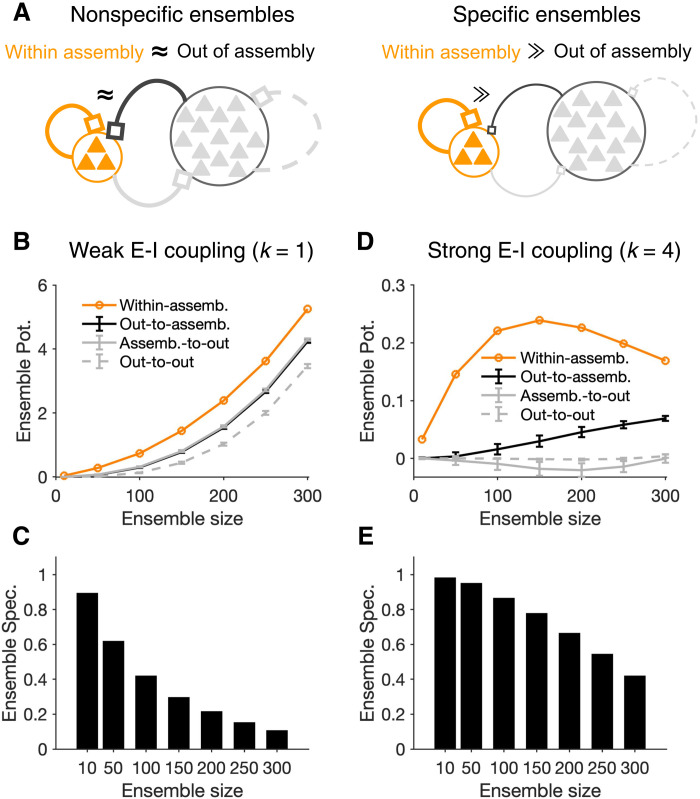
Specificity of assembly formation in different regimes of E/I balance. (**A**) The outcome of induction can be nonspecific (left), if the within-assembly potentiation of weights is accompanied by a substantial potentiation of connections originating from outside the perturbed ensemble, or specific (right), when the potentiation of weights remains constrained to the intended, perturbed ensemble. (**B**) Potentiation of presynaptic connections within the assembly (orange) versus those from the assembly to outside (assemb.-to-out; gray), from outside to the assembly (out-to-assemb.; black), and within the neurons outside the assembly (out-to-out; gray dashed), respectively. *T*_p_ = 50 and induction is in the weak E-I coupling regime (*k* = 1). Ensemble potentiation is calculated as the average (across postsynaptic neurons) of the sum of connection weights from all presynaptic sources (cf. Fig. 1J). For each *N*_p_, out-of-assembly potentiation is calculated for 100 randomly selected pools of neurons other than, but with the same size (*N*_p_) as, the perturbed neurons. Line and error bars show the average and SD across the pools, respectively. (**C**) Ensemble specificity (Spec.) quantifies the specificity of induced assemblies for different sizes of perturbed neurons. It is calculated as (*E*_w_ – *E*_o_)/(*E*_w_ + *E*_o_), where *E*_w_ and *E*_o_ are the average within- and out-of-assembly (assemb.-to-out) ensemble potentiation in (B), respectively. Ensemble specificity drops for larger ensemble sizes, reflecting the fact that within-assembly potentiation of weights is accompanied by a substantial potentiation of connections from outside. (**D** and **E**) Same as (B) and (C) for neuronal assemblies forming in networks with strong E-I coupling (*k* = 4). Out-of-assembly potentiation grows much slower than within-assembly potentiation initially until the latter plateaus and starts to drop (D), leading to a higher ensemble specificity for all ensemble sizes (E).

Our results so far indicated that cooperativity in the formation of neuronal assemblies emerges in networks with weaker E-I couplings and that this changes to suppressive effects in networks with stronger E-I interactions. Neuronal assemblies are thus expected to emerge faster and stronger in the former regime compared to the latter. However, how selective would the outcome of the induction be in each regime? To answer this, we quantified the selectivity of assembly formation by comparing the strength of presynaptic connections to perturbed neurons arising from within and outside the perturbed ensemble (Fig. 3A). If the outcome of induction is selective, then the potentiation of weights should remain confined to connections within the perturbed ensemble (within-assembly potentiation). On the other hand, correlated patterns of activity resulting from perturbations may also lead to potentiation of synapses from (or to) outside the perturbed ensemble, thus creating a degree of nonspecific, out-of-assembly potentiation.

For networks with weak E-I coupling, strong within-assembly potentiation was accompanied by a substantial out-of-assembly potentiation of weights, resulting in a substantial drop in specificity of assembly formation for large sizes of perturbed ensembles (Fig. 3, B and C). However, potentiation of connections from outside the ensemble grew much slower for networks with strong E-I coupling, leading to an optimal size of induction where the strongest within-assembly potentiation had also a high induction specificity (Fig. 3, D and E). We observed qualitatively similar results for different variants of the Hebbian plasticity rule (fig. S5). These results show that stronger potentiation of assemblies in networks with weak E-I coupling comes at the price of losing the specificity of induction, as the relative potentiation of within-assembly to out-of-assembly weights decreases for larger perturbed ensembles. Strong E-I interaction hampers the potentiation of synapses within perturbed ensembles but leads to a more selective formation of neuronal assemblies, which is more robust to changes in the size of perturbed ensembles.

Preexisting wiring in the network may guide the process of induction (*34*) and lead to a nonrandom distribution of weight changes. Connections between neurons are, in fact, reported to be organized according to their functional properties (*17*, *18*, *35*, *36*). We therefore asked how the modulation of out-of-assembly connections depends on the initial network structure in networks with some nonrandom (specific) connectivity structure. Specific connectivity was implemented by modulating the connection weights in the network to have stronger connections between pairs of neurons with similar functional properties, which was assumed to be a one-dimensional (1D) feature (e.g., preferred orientation) here (see Materials and Methods for details). Note that neuronal features are only used to generate the feature-specific connectivity in the network, but the activity of the network is simulated in the spontaneous state, where the input to the neurons is not modulated by these features.

In weak E/I regime (*k* = 1), we found feature-specific potentiation, namely, out-of-assembly connections potentiated more for neurons with similar functional features as the perturbed ensemble (fig. S6A). This result suggests that neuronal assemblies can recruit neurons with similar features out of the perturbed ensemble but also indicates that preexisting connectivity can interfere with within-assembly potentiation in this regime. For networks with strong E/I interactions (*k* = 4), on the other hand, we observed an opposite trend: Neurons closer in the functional space experienced, on average, a larger depression of their weights to the perturbed ensemble (fig. S6B). Such feature-specific depression of weights can increase the specificity of induction by suppressing the strong preexisting connections that might not be relevant to another stimulus. Different regimes of E/I can therefore support different modes of assembly formation with regard to preexisting structure of the network with weak E-I coupling regimes promoting the influence of the previous connectivity and strong E-I coupling enabling a more efficient “rewriting” of the circuitry.

Both feature-specific potentiation and depression were absent when initially perturbed neurons were chosen randomly, independent of their preferred orientations (fig. S6, A and B). These results therefore argue that different regimes of recurrent interaction as well as different patterns of induction can lead to distinct outcomes of plasticity. Note that, while we assumed similar properties for all neurons, accounting for more biologically realistic receptive fields of E/I neurons (*29*) and their connections [e.g., broader selectivity and connectivity of inhibition (*37*–*40*)] could lead to a center-surround pattern of out-of-assembly plasticity with potentiation and depression for highly similar and less similar connections, respectively [vis-a-vis center-surround patterns of influence resulting from neuronal perturbations in the visual cortex (*16*, *29*)].

Cortical networks regulate their activity following sensory deprivation (e.g., as a result of injury or input deprivation), and neuronal assemblies are suggested to be involved in subnetwork-specific recovery of such responses (*41*). We asked how this process can be guided by specificity of resulting assemblies in different regimes. We reduced the feedforward input to a fraction of neurons in the network (comprising distinct subnetworks, A and B) and studied how correlated external activation of a subset of them (subnetwork A) can lead to recovery. In both regimes, neurons in subnetwork A potentiated their recurrent weights, which can counteract the lack of feedforward drive after input deprivation (fig. S6, C and D). While this potentiation happened exclusively within subnetwork A in networks with strong E-I coupling (fig. S6D), recovery in weak E-I coupling regimes was also accompanied by potentiation of connections from other E neurons (fig. S6C). Specifically, the reciprocal connectivity between subnetworks A and B was potentiated in the weak E-I regime, while it was depressed in the strong E-I regime (fig. S6, C and D). These results therefore suggest that strong E-I interactions can shape the specificity of formation of neuronal assemblies in the network and their subsequent recovery following input deprivation.

### Speed and specificity of assembly formation

**Fig. 4.**
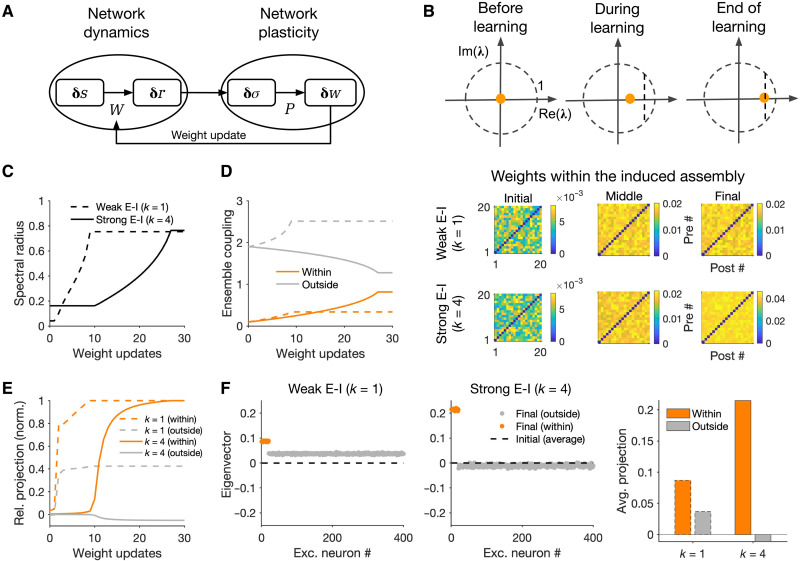
Growth of ensembles in networks with recurrent interaction of dynamics and plasticity. (**A**) Closed-loop interaction of network dynamics and network plasticity underlying the formation and growth of neuronal assemblies. Network dynamics governed by the weight matrix (*W*) determines the input-output responses to external perturbations, which, in turn, shape the structure of covariances. Network plasticity (*P*) guided by the resulting covariance patterns determines the weight changes and updates, on a slower time scale, the weight matrix, which, in turn, modifies the network dynamics. (**B**) Top: Spectral radius of the network denoting the growth of the maximum eigenvalue of the weight matrix (λ_0_) at different steps of weight update. To avoid instability of the network dynamics (λ_0_ > 1), the learning is stopped before λ_0_ reaches a threshold close to 1 (vertical dashed line). Bottom: Sample weight matrices of the perturbed ensemble at different stages for networks in different E/I regimes. *N*_p_ = 20, *T*_p_ = 50; other parameters the same as in Fig. 2. (**C**) Evolution of the spectral radius in different regimes. (**D**) Ensemble coupling (mean-field coupling of the populations) within the perturbed ensemble (orange) and from neurons outside the perturbed ensemble to the ensemble (gray) (cf. Fig. 3, B and D) at different weight updates (dashed, *k* = 1; solid, *k* = 4). (**E**) Relative projection of the eigenvector (*v*_0_) corresponding to the largest eigenvalue (λ_0_) of the network over neurons within (orange) and outside (gray) the perturbed ensemble for networks with *k* = 1 (dashed) and *k* = 4 (solid). It is calculated as the average real part of the entries corresponding to perturbed and nonperturbed neurons normalized by the maximum value for each regime. (**F**) Left: Distribution of the real part of the largest eigenvector (*v*_0_) over excitatory neurons at the end of learning. Dashed line, the average value (across excitatory neurons) of the initial distribution before induction. Right: Average projection of the final eigenvector over excitatory neurons within and outside the perturbed ensemble.

In the previous analyses, we focused on how response changes resulting from perturbations in different dynamic regimes guide network-wide plasticity and formation of assemblies (Fig. 1C). These unidirectional effects of dynamics on plasticity might, in fact, be pertinent to initial stages of the development of assemblies; in later stages, however, weight changes would, in turn, shape the network dynamics (although with a slower time course). To fully analyze the dynamic evolution and growth of neuronal assemblies in cortical networks, we therefore need to consider this closed-loop interaction of dynamics and plasticity (Fig. 4A).

To study this, we repeated our previous perturbation protocols while updating the weight matrix of the network in incremental steps. The weight matrix was updated in time intervals of Δ*T_w_*, while between the updates the weight matrix (*W*) was kept constant and determined the network dynamics. Note that Δ*T_w_* is much larger than the time scale of network integration (τ), which is justified by the separation of time scales of dynamics and plasticity (*42*). We used a Hebbian rate–based covariance rule to update the weights (see Materials and Methods). To ensure the stability of network dynamics, we perform the weight update at each stage only if the largest eigenvalue of the weight matrix (or its spectral radius) does not grow more than a value close to, but smaller than, 1 (Fig. 4B; Materials and Methods). Different mechanisms can be used to ensure such stability, e.g., hard bounds for the weights, weight normalization, synaptic scaling, or inhibitory stabilization (*32*, *43*, *44*), but our analysis here remains agnostic about the nature of this mechanism.

Growth of the spectral radius provides a proxy for the speed of learning in different regimes (Fig. 4C). The spectral radius grew much faster for networks with weak E-I coupling (*k* = 1), indicating a faster strengthening of weights in this regime. Evolution of the spectral radius was similar to the fast strengthening of weights within the perturbed ensemble in this regime (Fig. 4D). The fast growth of assembly was, however, accompanied with the fast potentiation of out-of-assembly connections (Fig. 4D). The evolution of neuronal assemblies in the strong E-I coupling regime (*k* = 4), on the other hand, was slow and specific: Both the spectral radius and the within-assembly weights grew much slower, but this was accompanied by weakening of connections from outside the perturbed ensemble leading to the specificity of assembly formation (Fig. 4, C and D).

Different patterns of growth of neuronal assemblies in different regimes can be explained in terms of the eigenvector corresponding to the largest eigenvalue of the network at each stage (Fig. 4, E and F). If mainly the connections within the perturbed ensemble are potentiated over time, then the eigenvector will have strong projections over the perturbed neurons with weak or zero contributions from other neurons. Nonspecific growth would, on the other hand, translate to a more even projection of the eigenvector over perturbed and unperturbed neurons. Networks with strong E-I couplings showed specific projections, while the selectivity of projection was much lower for networks with weak E-I coupling during learning (Fig. 4E) and at the end of it (Fig. 4F). Thus, although the largest eigenvalue of the network grows faster in weak E-I regimes (Fig. 4C), its corresponding eigenvector does not remain confined to perturbed neurons (Fig. 4E), indicating that within-assembly potentiation of weights is accompanied by potentiation of connections from outside the perturbed ensemble (Fig. 4D). The growth of eigenvalue in strongly coupled E-I regimes is slower, but the corresponding eigenvector and the potentiation of weights remain specific to perturbed neurons, ensuring a selective formation of assemblies (Fig. 4, C to F).

### Pattern completion and different regimes of recall

**Fig. 5.**
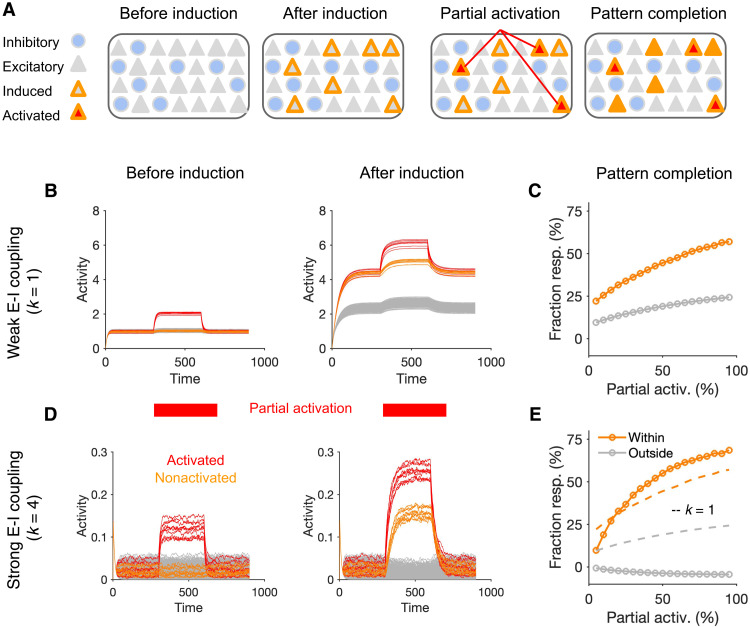
Pattern completion in neuronal ensembles emerging in different E/I regimes. (**A**) Pattern completion is triggered by partial activation of neurons within an induced assembly and evaluating the response of other nonactivated neurons. (**B**) Response of the network with weak E-I coupling (*k* = 1) in the baseline with and without partial activation of the assembly. Left: Before the formation of assembly (before induction). Right: After induction with updated weights at the end of learning, as described in Fig. 4. Half of the neurons in the induced assembly (10 of 20, shown in red) are stimulated by extra perturbations, and the effect on other excitatory neurons within (orange) and outside (gray) the assembly is evaluated. (**C**) Pattern completion curve describing the degree of pattern completion (quantified by Fraction resp.) as a result of partial activation of the assembly (Partial activ.). Fraction resp. is calculated as the average response change of the nonactivated neurons [within (orange) and outside (gray) the assembly, respectively] divided by the average response change of activated neurons. Response changes are measured relative to the respective baseline activity of each neuron before partial activation. Note that this is a conservative measure for quantifying pattern completion, which only reaches 100% when all nonactivated neurons reach the same level of activity as activated ones. (**D** and **E**) Same as (B) and (C) for networks with strong E-I coupling (*k* = 4). The pattern completion curve for *k* = 1 is copied in (E) for comparison (dashed lines). The strength of external perturbations is larger for *k* = 1 to adjust for the higher baseline activity of the network (see Materials and Methods for details).

A suggested functional benefit of neuronal assemblies is their capacity to amplify weak signals, to reduce the noise in the input, and to activate a stored representation when only a fraction of the pattern is presented to the network (pattern completion). We therefore studied how neuronal assemblies emerging in each regime show pattern completion (Fig. 5). At the end of learning (Fig. 4B), we partially activated the neurons within the perturbed ensemble and measured the response of other neurons within the ensemble that were not directly activated by the external stimulation (Fig. 5A). Neuronal assemblies formed in both regimes showed pattern completion when half of their neurons were activated (Fig. 5, B and D). We further quantified the strength of pattern completion for different fractions of partial activation. This was calculated by comparing the average response of the nonactivated (NA) and activated neurons within the perturbed ensemble (see Materials and Methods). Both networks showed comparable pattern completion curves within the ensemble with even small fractions of activation eliciting notable responses in NA neurons (Fig. 5, C and E).

However, pattern completion was more constrained to the targeted assembly in networks with stronger E-I couplings. In the network with strong E-I coupling (*k* = 4), only NA neurons within the perturbed ensemble were activated as a result of recurrent interactions (Fig. 5E). In contrast, in the network with weaker E-I coupling (*k* = 1), recurrent interactions also elevated the activity of neurons outside the assembly, leading to some degree of off-target pattern completion (Fig. 5, B and C). Together, these results suggest that neuronal assemblies forming in networks with weak or strong E-I coupling may enable computations with different speed-accuracy trade-off and can therefore contribute differently to cortical processing.

### Behavioral performance associated with neuronal assemblies in different E/I regimes

**Fig. 6.**
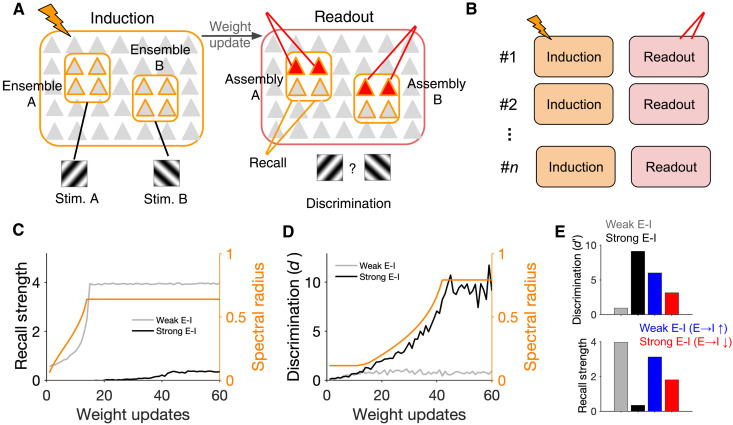
Performance of neuronal assemblies in behavioral tasks in different regimes of recall. (**A**) Left: In networks with different regimes of E-I coupling, two neuronal ensembles (A and B), each representing a specific stimulus (stim. A and B, respectively), are perturbed during induction to form neuronal assemblies. Right: After each weight update, performance of the network is evaluated by a downstream decoder that reads out the responses of assemblies to partial triggers of their neurons. (**B**) The readouts are evaluated after each induction session, and the weights are updated before the next induction, following the same procedure described in Fig. 4. *N*_E_ = *N*_I_ = 200. (**C**) Recall strength is quantified after each weight update to evaluate the capacity of the formed assembly (*N*_p_ = 20) to detect its associated stimulus. It is calculated as the average increase in the activity of nontriggered neurons (15 of 20) in the assembly, when a small fraction of neurons (5 of 20) are stimulated. The evolution of the spectral radius in the weak E-I regime (*k* = 1) is plotted on the right *y* axis for comparison. (**D**) A discriminability index is calculated at each readout session to evaluate the capacity of the assemblies to distinguish between the two stimuli (see Materials and Methods). The evolution of the spectral radius in the strong E-I regime (*k* = 4) is plotted on the right *y* axis for comparison. (**E**) Discrimination and recall strength for networks with weak E-I (gray) and strong E-I (black) coupling at the end of learning. The results are compared with networks with weak E-I coupling when the recruitment of inhibition is boosted via E➔I connections (inhibitory modulation; blue) and to networks with strong E-I coupling where E➔I connections are weakened (disinhibitory modulation; red). These modulations enhance discriminability in networks with weak E-I and increase the recall strength in networks with strong E-I coupling regimes, respectively.

Once formed, neuronal assemblies associated with different stimuli can guide and trigger the behavior (e.g., avoiding the aversive stimuli in fear-conditioning tasks or choosing the target stimuli to get the reward). We therefore asked how neuronal assemblies forming in different E/I regimes can contribute to behavioral performance by simulating the development of two neuronal assemblies (A and B) associated with two distinct stimuli (Fig. 6A, left). The association was established in induction sessions, where two nonoverlapping ensembles of neurons were perturbed in an alternating way (similar to protocols described in Fig. 4; *N*_p_ = 20 for each perturbed ensemble). The weight matrix of the network was updated at the end of each induction session on the basis of the covariance of activity resulting from both perturbation patterns. The behavioral performance was then assessed in recall sessions (Fig. 6B), where a fraction of neurons (5 of 20) in each assembly (A or B, respectively) was stimulated. The capacity of the network to detect the presence of a stimulus was assayed by quantifying the “recall strength” of the respective assembly (see Materials and Methods). The performance of the network to distinguish between the two stimuli was quantified by calculating a “discriminability index” (*d*′), which compared the response of a given assembly to its associated and irrelevant stimuli (see Materials and Methods for details).

Networks with weak E-I coupling showed a very swift increase in recall strength, which matched with the quick growth of their spectral radius (Fig. 6C). This shows that neuronal assemblies in this regime can amplify a weak stimulation of a small fraction of their neurons providing a substrate for fast and strong recalls. In comparison, recall strength was much weaker and rose up much more slowly in networks with strong E-I coupling (Fig. 6C). Neuronal assemblies in the latter regime had, however, a clear advantage in discriminating between the two stimuli (Fig. 6D). While the initial enhancement of discriminability (*d*′) plateaued in weak E-I regimes, neuronal assemblies in strong E-I regimes improved their discrimination capacity for much longer and to much higher values, matching the slower growth of their spectral radius (Fig. 6D).

These results suggest that neuronal assemblies emerge slower in inhibition-dominated regimes and can enable fine discriminations in downstream decoders (like distinguishing between two gratings with slightly different orientations), while the assemblies forming in weaker E-I regimes can be suited for faster but more crude cognitive tasks (e.g., detecting the movement of a looming stimulus mimicking the predator). Modulating E-I balance in the network, for instance, by top-down mechanisms [e.g., via vasoactive intestinal peptide (VIP)–positive neurons] (*45*–*47*) can therefore provide a powerful tool to control different modes of learning (*48*). We tested this in our networks and found that modulating E➔I coupling bidirectionally modulated learning: Increasing E➔I coupling in weak E-I networks increased discrimination (and decreased the recall strength), while decreasing E➔I coupling in strong E-I networks increased recall strength (and reduced discrimination) (Fig. 6E). Different modes of learning and induction can therefore be achieved by general modulation of the network.

### Dynamic transition between different regimes resulting from E-I plasticity

In our networks so far, we only allowed E-E synapses to be plastic and studied the effect of E-I interactions on this plasticity by changing static E-I weights in different E/I regimes. These regimes may not be static, however, and can be dynamically modulated, as we discussed in the previous section. In addition to external mechanism like top-down modulation, plasticity of E-I connections within the network can also intrinsically change the E-I coupling (*49*, *50*). Different plasticity rules of subtypes of inhibitory neurons indeed shape dynamics and learning in different manners (*51*–*53*). We therefore studied how E-I plasticity may contribute to the regimes of induction by extending our model networks: In addition to E-E synapse, we now allowed E-I (E➔I and I➔E) synapses too to be governed by covariance-based Hebbian rules (cf. Fig. 4).

**Fig. 7.**
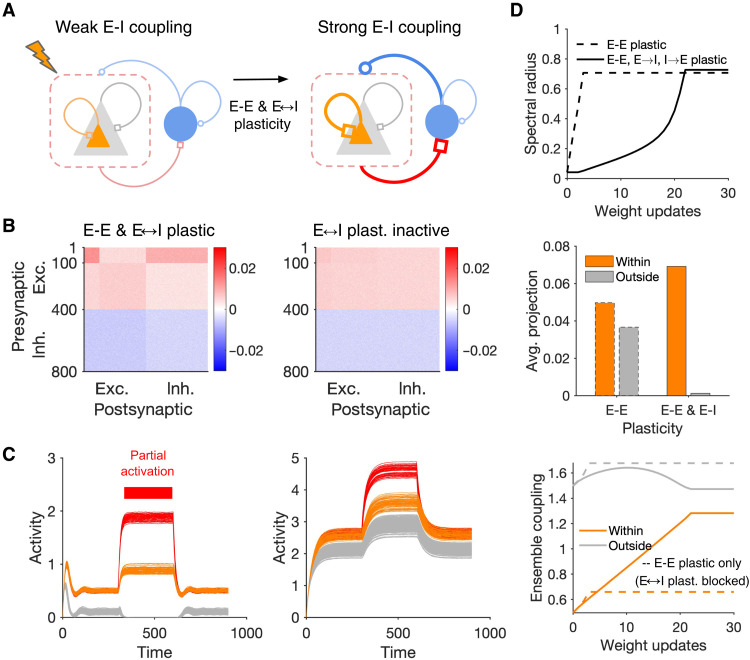
Dynamic transitions between different regimes of assembly formation. (**A**) Schematic of a network with E-E and E-I plasticity before and after induction of assemblies. (**B**) Final weight matrix of the network at the end of learning (similar to the procedure described in Fig. 4) in the network where both E-E and E-I weights are plastic (left) compared with the condition where E-I plasticity is blocked and only E-E plasticity remains (right). *N*_E_ = *N*_I_ = 400, *N*_p_ = 100 (perturbed neurons #1 to 100). (**C**) Pattern completion in networks with E-E and E-I plasticity (left) and only E-E plasticity (right) at the end of learning. Note that different levels of baseline activity in Figs. 5 and 7 is a result of different sizes of perturbed ensembles (*N*_p_ = 20 versus 100, respectively). (**D**) Growth of the spectral radius (top; cf. Fig. 4c), average projection of the largest eigenvector over excitatory neurons (middle; cf. Fig. 4F), and evolution of ensemble coupling (bottom; cf. Fig. 4D) in the networks with E-E and E-I plasticity (solid lines) and when E-I plasticity is blocked (dashed lines).

Combining E-E and E-I plasticity can enable the perturbed ensemble of neurons to strengthen their E-I interactions (Fig. 7A). Induction of neuronal assemblies in networks with E-E and E-I plasticity indeed led to a strong potentiation of perturbed excitatory neurons (Fig. 7B, left); this potentiation was much weaker when E-I plasticity was inactive (Fig. 7, B, right and D). Potentiation of perturbed E-E ensembles was anticorrelated with the average activity of the networks: Networks with E-E and E-I plasticity had smaller baseline activity, while the baseline activity increased for networks in which E-I plasticity was inactive (Fig. 7C). Sparsification of activity in the network with E-E and E-I plasticity was a result of potentiation of E-I coupling, which put the network in a more inhibition-dominated regime (*50*).

The network with E-E and E-I plasticity showed selective pattern completion upon partial activation of neurons in the perturbed ensemble (Fig. 7C, left), and this selectivity was diminished in networks without E-I plasticity (Fig. 7C, right) (cf. Fig. 5, B and D). Slow but selective growth of the eigenvector associated with the largest eigenvalue shed light on the slow and selective potentiation of within-assembly weights, which was in contrast to fast and nonspecific formation of assemblies when E-I plasticity was blocked (Fig. 7D; cf. Fig. 4, C to F, for different E-I regimes). Our results held for another implementation of E-I plasticity based on presynaptic covariances and for different sizes of perturbed ensembles (fig. S7). Together, these results suggest that E-I plasticity can enable the network to dynamically transition between different regimes of induction and learning.

## DISCUSSION

We studied how different patterns of perturbations can induce neuronal assemblies in large-scale balanced networks. Our results revealed different regimes of induction for the spectrum of excitation-inhibition balance. In particular, we found that increasing the size of perturbed ensembles may not always lead to more potentiation. Induced assemblies in regimes with dominant E-I coupling exert a potent lateral inhibition, which suppresses the activity of neurons and the potentiation of their respective connections. This would also apply to connections within the perturbed ensemble in a recurrent manner, leading to a sublinear growth of the targeted assemblies. Although hampering the general strength of plasticity, recurrent inhibition increases the specificity of assembly formation by suppressing off-target potentiation of synapses.

Our results suggest that inhibition can gate and modulate the speed and specificity of induction. Inhibition dominance slows down the formation and growth of neuronal assemblies but ensures that perturbation-induced plasticity remains constrained to perturbed ensembles. While the selectivity of resulting assemblies quickly vanished in weak E-I regimes for larger sizes of perturbed ensembles, this selectivity remained more robust for networks with dominant E-I coupling, suggesting that inhibition can also contribute to size-invariant selectivity of assembly formation. These inhibition-dominated regimes were, in turn, best suited for fine-discrimination tasks, which rely on selectivity of neuronal assemblies. It would be interesting to test these predictions in future experiments by modulating E-I balance (*54*) in cortical networks and measuring the specificity of assembly formation and behavioral discriminability.

When excitation was predominant, induction was fast and strong in our network models but did not remain constrained to the perturbed ensemble. Because of an indirect recurrent recruitment of other neurons in the network, connections from/to outside the targeted assembly also strengthened. This regime can thus enable recruitment of other neurons to expand the representations. However, it compromises the selectivity of pattern completion by neuronal assemblies (i.e., activating NA neurons inside as well as outside the targeted assembly) and reduces the capacity of the network to discriminate between stimuli represented by different assemblies. This regime might therefore be better suited for crude detection tasks and can provide a substrate for generalization to other assemblies and beyond a given stimulus.

Which regimes of induction are more pertinent to the regimes in which cortical networks operate? Functionally, network with strong E-I coupling can provide a natural substrate for recent behavioral findings, which suggest that mutual inhibition of neuronal assemblies can underlie their selective responses (*55*, *56*). In terms of connectivity, the key ingredient of the strong E-I coupling regime has been observed in many cortical regions, where a dense and strong connectivity between pyramidal cells (PCs) and different subtypes of interneurons, including parvalbumin-positive (PV^+^) and somatostatin-positive (SOM^+^) cells, has been reported (*57*, *58*). In the mouse primary visual cortex (V1), for instance, both PC-to-PV and PV-to-PC connections are an order of magnitude larger than PC-PC connections (*59*). This regime is also consistent, in terms of dynamics, with recent results from single-neuron optogenetic studies (*16*): The prevalence of suppressive effects reported in the experiments only emerges in networks with dominant E-I coupling (*29*). It is therefore likely that mouse V1 operates in an inhibition-dominated regime that favors selective (but weak and slow) induction of assemblies.

This regime might be relevant to other cortices, and species, too. Inhibition-stabilized regimes are believed to be even more pronounced in other species like cats and monkeys with strong recurrent interactions (*60*–*62*). Strong local excitatory-inhibitory coupling has also been reported in other areas, including mouse somatosensory and frontal cortex (*57*, *58*, *63*, *64*). In the mouse barrel cortex, optogenetic stimulation of ~100 excitatory neurons induced a strong inhibition of neighboring excitatory neurons, arguing for an inhibition-dominated regime of activity favoring competition and sparsification (*63*). Optogenetic stimulation induced rapid excitation (at ~5 ms), which was quickly quenched by inhibition (at ~10 ms) (*63*), consistent with our results on the emergence of suppressive effects for longer pulses (*T*_p_) (cf. Fig. 1J and fig. S4). It would be interesting to explore in future studies how different combinations of stimulation protocols and operating regimes contribute to formation of neuronal assemblies in different cortices.

The operating regime of induction can, in turn, be dynamically modulated across different cortices and layers by different factors, including behavioral states [e.g., transition from anesthetized to awake states (*65*) or stationary versus running (*45*)], neuromodulation, and attention. For instance, top-down inputs can disinhibit the local circuitry (via VIP➔SOM disinhibition) (*45*), VIP neurons can control different stages of learning by differential recruitment of PV neurons (*48*), and the neuromodulatory suppression of PV cells by SOM neurons is crucial for the onset and closure of the critical period of plasticity (*66*). Our results too suggest that modulating E-I coupling—either via top-down modulation (Fig. 6) or by plasticity of E-I interactions (Fig. 7)—can provide a potent mechanism to control the formation of neuronal assemblies in different regimes of learning. Plasticity of E➔I connections has indeed been shown to be crucial for gating of memory and network plasticity (cf. Fig. 7) (*67*). On the other hand, different inhibitory subtypes show different patterns of plasticity, with PV and SOM neurons undergoing depression and potentiation of their weights, respectively, in response to sustained activation (*51*–*53*). SOM neurons might therefore be in a key position to dynamically gate the plasticity and transition to and from strong E-I coupling regimes. It would be interesting to see, in future studies, if and how bidirectional manipulation of these mechanisms can modulate the formation of neuronal assemblies in different regimes.

Future theoretical work is also needed to study different regimes of induction in more realistic conditions. First, following the classical notion of neuronal assemblies as recurrent subnetworks (namely, subset of neurons with highly recurrent interconnectivity), we focused our analysis on recurrent dynamics and the resulting weight changes in recurrent connections after perturbations. However, external perturbations may also affect the plasticity of feedforward weights or excitability of the neuron. In particular, it would be interesting to see how the plasticity of feedforward synapses in the evoked state interacts with recurrent connections (*68*). These evoked states will also amplify the effect of stimulus selectivity of neurons and hence their preexisting connectivity based on that selectivity, which might, in turn, guide or limit the induction (*34*). Extension of our model networks to allow for feedforward and recurrent plasticity can shed light on these more realistic regimes of network responses and plasticity.

Network models studied here mainly focused on the effect of recurrent E-I interactions on the average rate of spiking, both in terms of network activity (rate-based) and plasticity rules (covariance-based). However, E/I balance can also affect precise temporal dynamics of network responses (*30*), and inhibitory neurons are known to control timing as well as rate of spiking (*69*). Temporal correlations emerging in spiking networks (*70*) especially in excitation-dominant regimes (*71*, *72*) may, for instance, amplify global potentiation across the network when spike time–dependent plasticity (STDP) rules are used. Biologically, however, it seems that both spike timing and spike frequency matter for plasticity, and rules based on a combination of voltage and spike timing have been shown to fit experimental data better than the standard STDP rule (*73*, *74*). We could, in fact, show that our simulations yield similar results with a voltage-based STDP rule, which depends on both spiking timing and activity- or voltage-based traces (fig. S3, E and F). Future extension of our theoretical analysis is needed to account for more precise timing of spiking activity in combination with rate-based parameters.

To obtain computational insights into the basic properties of network-wide plasticity and assembly formation, we focused our analysis on simple models of dynamics and plasticity. Inevitably, many biological mechanisms were absent from our models. It would therefore be important to investigate our results in more realistic networks, including those with more complex single-cell mechanisms like dendritic nonlinearity and plateau potentials, as well as networks equipped with other rules of plasticity (*49*, *50*, *75*) and homeostasis (*44*, *76*). It would also be interesting to study how different subtypes of inhibition and their mutual disinhibition (*77*) affect our results, as well as various rules of inhibitory plasticity (*51*, *53*, *78*) associated with them.

In summary, our work highlights the importance of studying dynamics of neuronal networks and network-wide plasticity in tandem to cast light on the formation of neuronal assemblies. It suggests that unexpected results may emerge when considering the recurrent interactions within networks of excitatory and inhibitory neurons and that these effects might be missed by focusing on isolated pairs of neurons detached from their network interactions. As behaviorally relevant learning is ultimately happening in ensembles of neurons embedded in large-scale recurrent networks, it is crucial to understand the effect of the background dynamics on the formation of neuronal assemblies and learning. Here, we developed a computational framework to help with this understanding, which can guide the design of future perturbation protocols.

## MATERIALS AND METHODS

### Network simulations

Rate-based networks were simulated by the following equations for excitatory (E) and inhibitory (I) neurons

$$\begin{array}{c} \tau \frac{dr_{E}}{\mathrm{dt}}=-r_{E}+{\left[W_{\text{EE}}r_{E}+W_{\text{EI}}r_{I}+s_{E}\right]}_{+}\\ \tau \frac{dr_{I}}{\mathrm{dt}}=-r_{I}+{\left[W_{\text{IE}}r_{E}+W_{\text{II}}r_{I}+s_{I}\right]}_{+} \end{array}$$ (1)

where *r*_E_ and *r*_I_ are the vectors of firing rates of E and I neurons and *s* is the external input with *s*_E_ and *s*_I_ denoting inputs to E and I neurons, respectively. In the baseline state, before perturbations, all neurons in the network receive a baseline input *s*_0_ + ζ, where *s*_0_ = 1 and ζ is drawn from a uniform distribution between [0,0.1]. *W* is the matrix of connection weights, including connections between E-to-E (*W*_EE_), E-to-I (*W*_IE_), I-to-E (*W*_EI_), and I-to-I (*W*_II_) neurons. τ is the effective time constant of the network integration, and []_+_ denotes half-wave rectification. We used the forward Euler method to solve for the firing rates of neurons. Regime of network connectivity was parameterized by the relative strength of average coupling between different subpopulations *J*_IE_=∣*J*_EI_∣=∣*J*_II_∣= *kJ*_EE_ (Fig. 1G). Here, *J_YX_* denotes the average coupling from population *X* to population *Y* ({*X*, *Y*} ∈ {*E*, *I*}), and it is obtained as the average (over postsynaptic neurons) of the sum of weights from presynaptic sources: $J_{\mathrm{YX}}=\frac{1}{N_{Y}}\sum_{j\in X}\sum_{i\in Y}w_{\mathrm{ij}}$, where *N_Y_* is the size of postsynaptic population and w*_ij_* is the weight of connection from *j*th to *i*th neuron.

Spiking networks were modeled by simulating the equations describing the membrane potential dynamics of leaky integrate-and-fire neurons

$$\tau_{m}\frac{dV_{m}}{\mathrm{dt}}=-V_{m}(t)+s(t)$$ (2)

where *V*_m_ is the membrane potential of a neuron and τ_m_ = *RC* is the time constant of integration of the membrane potential with *R* and *C* denoting the membrane resistance and capacitance, respectively. When the membrane potential reaches a voltage threshold (*V*_th_), a spike is elicited and the membrane potential is reset to the reset voltage, *V*_reset_ = 0. *s*(*t*) = *RI*(*t*) describes the momentary input to the neurons, which arises from incoming spikes and comprises external (feedforward and nonlocal) input and recurrent input from presynaptic neurons in the network. Once a spike is emitted in a presynaptic source, an instantaneous change in the membrane potential of all postsynaptic sources is emulated in the next simulation time step, by the value of *w*, which is expressed in units of volts and describes the effect of *RI* simultaneously. The total input at time *t* for a postsynaptic neuron *i* is given by *s*(*t*) = Σ*_j_ w_ij_*δ*_j_*(*t*), where δ*_j_*(*t*) denotes the presence (1) or absence (0) of spike in presynaptic sources, with *w_ij_* describing the weight of connection from the *j*th presynaptic source. We used the exact integration method (*79*) to solve for the membrane potential and spiking activity of neurons.

Network connectivity is described by the weight matrix *W*, with *w_ij_* denoting an entry on its *i*th row and *j*th column. Connections between neurons are established by drawing from a binomial distribution with probability ϵ (ϵ = 1 for networks with all-to-all connectivity). *w_ij_* is set to zero if there is no connection from a pre- to postsynaptic neuron. If there is a connection, *w_ij_* is drawn from a uniform distribution (with mean *J*) in randomly connected networks. In networks with nonrandom (specific) recurrent connectivity, *w_ij_* depends on the functional similarity of pre- and postsynaptic neurons. Neurons are assumed to have a 1D receptive field (e.g., orientation selectivity), and the weight of connections is modulated as

$$w_{\mathrm{ij}}=J\;(1+m\;\text{cos}(2(\theta_{i}^{*}-\theta_{j}^{*})))$$ (3)

where θ_i_^*^ and θ_j_^*^ are the preferred orientation (in radians) of pre- and postsynaptic neurons, which are drawn randomly from the range [0, π), and *m* controls the degree of specificity of the connections (with *m* = 0 recapitulating random, nonspecific connectivity). Default parameters of simulations are listed in Table 1.

**Table 1.** Table of parameters.

**Description**	**Type**	**Parameter**	****Fig. 1****	**Figs. 2 and 3**	**Figs. 4 and 5**	****Fig. 6****	****Fig. 7****
No. of neurons	E	*N*_E_	500	500	400	200	400
	I	*N*_I_	500	500	400	200	400
Time constant of neuronal integration	E and I	τ	10	10	10	10	10
Average weight of synaptic connections	E → E	*w*_EE_	0.004	0.004	0.005	0.005	0.005
	E → I	*w*_IE_	0.004 (*k* = 1)	0.004 (*k* = 1)	0.005 (*k* = 1)	0.005 (*k* = 1)	0.005 (*k* = 1)
			0.016 (*k* = 4)	0.016 (*k* = 4)	0.02 (*k* = 4)	0.02 (*k* = 4)	0.02 (*k* = 4)
	I → E	*w*_EI_	−0.004 (*k* = 1)	−0.004 (*k* = 1)	−0.005 (*k* = 1)	−0.005 (*k* = 1)	−0.005 (*k* = 1)
			−0.016 (*k* = 4)	−0.016 (*k* = 4)	−0.02 (*k* = 4)	−0.02 (*k* = 4)	−0.02 (*k* = 4)
	I → I	*w*_II_	−0.004 (*k* = 1)	−0.004 (*k* = 1)	−0.005 (*k* = 1)	−0.005 (*k* = 1)	−0.005 (*k* = 1)
			−0.016 (*k* = 4)	−0.016 (*k* = 4)	−0.02 (*k* = 4)	−0.02 (*k* = 4)	−0.02 (*k* = 4)
Connection probability	All	ϵ	1	1	1	1	1
Synaptic specificity	All	*m*	0	0	0	0	0
No. of perturbed neurons	E	*N*_p_	10, 50, 100, 150, 200	10, 50, 100, 150, 200	20	20	100
Time of pulse on/off		*T*_p_=	10, 20, 50, 100	10, 20, 50, 100	50	100	50
		*T*_on_ = *T*_off_					
Stimulus (baseline)	E	*s*_0_	1	1	1	1	1
Perturbation strength	E	δ*s*	0.1 (*k* = 1)	0.1 (*k* = 1)	0.1 (*k* = 1)	0.1 (*k* = 1)	0.1 (*k* = 1)
			0.1 (*k* = 4)	0.1 (*k* = 4)	0.1 (*k* = 4)	0.1 (*k* = 4)	0.1 (*k* = 4)
Learning rate	E → E	η_EE_	–	–	0.2	0.1	0.05
	E → I	η_IE_	–	–	0	0	0.005
	I → E	η_EI_	–	–	0	0	0.005
	I → I	η_II_	–	–	0	0	0

### Network plasticity

To induce neuronal assemblies, a subset of *N*_p_ excitatory neurons in the network are perturbed (the perturbed ensemble). The perturbation pattern consists of *n_s_* alternating pulses (ON/OFF); each pulse stays ON (*s*_ON_ = *s*_0_ + δ*s*) for *T*_ON_ and turns off (*s*_OFF_ = *s*_0_) for *T*_OFF_. *s*_0_ describes the input to the neurons before perturbations, and δ*s* denotes the strength of perturbation [e.g., corresponding to laser intensity in optogenetic stimulations (*16*)]. The total duration of perturbation is therefore *n* (*T*_ON_ + *T*_OFF_) with the duty cycle of *T*_ON_/(*T*_ON_ + *T*_OFF_). Assuming *T*_p_ = *T*_ON_ = *T*_OFF_, the stimulation frequency is *f*_p_ = 1/*T*_p_.

Following perturbations, synaptic plasticity is assumed to change the initial weight matrix as a result of network activity. The change in the weight *w_ij_* is given as a function of the activity of pre- and postsynaptic neurons

$$\Delta w_{\mathrm{ij}}=\eta <(r_{j}-r_{j}^{0})\;(r_{i}-r_{i}^{0})>$$ (4)

where *r_j_* and *r_i_* describe the firing rate of pre- and postsynaptic neurons, respectively, η is the learning rate, and <. > denotes the temporal average that is evaluated during perturbations. *r*^0^ denotes the average firing rate of individual neurons in their baseline state obtained from network simulations before perturbations. We refer to this rule as covariance-based Hebbian learning, where covariance of the activity of pre- and postsynaptic neurons drives the plasticity. Two other versions of the rule are also tested, where response changes in only pre- or postsynaptic sources are considered, while the other term (post or pre, respectively) is still contributing to plasticity in absolute terms

$$\begin{array}{c} \text{pre}:\;\Delta w_{\mathrm{ij}}=\eta <(r_{j}-r_{j}^{0})\;r_{i}>\\ \text{post}:\;\Delta w_{\mathrm{ij}}=\eta <r_{j}\;(r_{i}-r_{i}^{0})> \end{array}$$ (5)

At each weight update, the weights of synapses are updated according to the following: *w_ij_* ← *w_ij_* + Δ*w_ij_*. After each update, we calculate the largest eigenvalue of the updated weight matrix (spectral radius; λ_0_). Weight update continues until the spectral radius is smaller than a threshold (λ_0_ < λ_th_ = 0.8); after that, the update stops and the last value of the weight matrix before passing the threshold is taken.

The learning rate (η) is chosen such that the growth is not too fast or too slow. If η is too small, too many update steps are needed to grow the assemblies, and simulations become computationally expensive. If η is too large, the spectral radius passes the threshold before any learning and assembly formation happen. In particular, the growth of the assemblies in the weak E-I regime can be very fast and spectral radius can undergo abrupt transitions to instability between consecutive updates, if the learning rate is not chosen properly. This choice also depends on the number of perturbed neurons: Larger sizes of perturbed ensemble (*N*_p_) lead to faster transitions to instability, as the potentiation happens within a bigger subnetwork. This rationale underlies the choice of different learning rates for different simulations, e.g., Fig. 5 (*N*_p_ = 20 and η = 0.2) versus Fig. 7 (*N*_p_ = 100 and η = 0.05).

Input deprivation was modeled by reducing the feedforward input to a fraction of neurons in the network. The baseline input (*s*_0_) to 200 excitatory neurons (#1 to 200) was reduced to half the initial value (*s*_0_/2). During recovery, correlated input patterns (similar to those delivered in the induction protocol; c.f. Fig. 1, with *T*_p_ = 50) are assumed to drive one of the subnetworks (subnetwork A: #1 to 100), while the other subnetwork (B: #101 to 200) does not receive the extra input. The recovery is assessed by evaluating ensemble potentiation of the two subnetworks (fig. S6, C and D).

### Data analysis

To quantify the strength of assembly formation as a result of perturbation-induced plasticity, we calculated ensemble potentiation as a measure of potentiation of synapses within the perturbed ensemble. For each postsynaptic neuron (*i*) in the targeted pool of neurons (Ω), the sum of weight changes from the presynaptic sources within the pool (*j* ∈ Ω) is calculated: ∑_*j* ∈ Ω_Δ*w_ij_*. Ensemble potentiation within the perturbed ensemble (*E*_w_) is then obtained as the average of this value over postsynaptic neurons within the pool (*i* ∈ Ω)

$$E_{w}=\frac{1}{N_{p}}\sum_{i\in \Omega }\sum_{j\in \Omega }\Delta w_{\mathrm{ij}}$$ (6)

Out-of-assembly potentiation is, in turn, quantified by the average (over postsynaptic neurons within the targeted pool: *i* ∈ Ω) of the sum of presynaptic weight changes from excitatory neurons outside the perturbed ensemble (*j* ∉ Ω)

$$E_{o}=\frac{1}{N_{p}}\sum_{i\in \Omega }\sum_{j\notin \Omega }\Delta w_{\mathrm{ij}}$$ (7)

Specificity of induction is quantified by comparing within-assembly and out-of-assembly potentiation: (*E*_w_ – *E*_o_)/(*E*_w_ + *E*_o_). To quantify the average weight changes within perturbed ensembles per individual synapse (e.g., as in Fig. 2 and as used in the “Theoretical analysis” section below), we also calculated average potentiation as: $\frac{1}{N_{p}^{2}}\sum_{i\in \Omega }\sum_{j\in \Omega }\Delta w_{\mathrm{ij}}$.

Task-specific performance of induced neuronal assemblies was quantified by two metrics (Fig. 6). First, recall strength was used to measure the absolute capacity of neuronal assemblies to trigger readout responses upon partial stimulation. The activity of the linear readout was calculated as the sum (over neurons) of the activity of NA cells in the assembly: *r*_ro_ = ∑_*i* ∈ NA_*r_i_*. The average (temporal) differential response of the readout after partial activation was taken as a measure of recall strength: $<r_{\text{ro}}-r_{\text{ro}}^{0}>$, where $r_{\text{ro}}^{0}$ is the baseline activity of the readout and <. > denotes temporal averaging evaluated during partial activation.

A discriminability index was also developed to characterize how neuronal assemblies can distinguish between different stimuli (Fig. 6, A and B). Using signal detection theory, it was calculated as

$$d\prime =(\mu_{s}-\mu_{n})/\sqrt{{\sigma_{s}}^{2}+{\sigma_{n}}^{2}}$$ (8)

where μ_s_ and μ_n_ are the average readout responses to “signal” (relevant stimulus) and “noise” (irrelevant stimuli), respectively. μ_s_ is calculated as the average (across repetitions) of <*r*_ro_>, when a small number of neurons in the same assembly are activated, while μ_n_ corresponds to the condition where same number of neurons from the other assembly are stimulated. σ is the SD of <*r*_ro_> over different repetitions of the partial stimulation in respective conditions.

### Theoretical analysis

To obtain theoretical insights into our numerical simulations, we analyzed how assemblies form in different E/I regimes and by different perturbation patterns. We first calculated the average potentiation of synapses expected from the linearized dynamics of the network. Writing Eq. 1 for the stationary state of network responses (*dr*/*dt* = 0), we have

$$r={\left(I-W\right)}^{-1}s$$ (9)

where s=(sEsI) and r=(rErI) denote the *N* × 1 vectors of input and output activity, respectively (with *N* denoting the total number of E and I neurons in the network, *N* = *N*_E_ + *N*_I_). Perturbation of a subset of excitatory neurons by δs=(δsE0) changes the output firing rates

$$\delta r={\left(I-W\right)}^{-1}\delta s$$ (10)

Applying the covariance-based Hebbian rule in Eq. 4, weight changes can be written as

$$\Delta w_{\mathrm{ij}}=\eta \;\delta r_{i}\delta r_{j}$$ (11)

Matrix of weight changes *P* with entry *p_ij_* = Δ*w_ij_* on *i*th row and *j*th column representing the weight change of the connection from the *i*th presynaptic neuron to the *j*th postsynaptic one can therefore be expressed as

$$P=\eta \;\delta r\delta r^{T}$$ (12)

where Σ = δ*r*δ*r^T^* is the covariance matrix of response changes following perturbations. Writing *A* = (*I* − *W*)^−1^ and substituting Eq. 10, matrix of plasticity *P* can, in turn, be expressed in terms of input perturbations as

$$P=\eta \;A\;\delta s\;\delta s^{T}A^{T}$$ (13)

For different patterns of perturbations of excitatory neurons with different *N*_p_ and strength of perturbation, we can evaluate the ensemble potentiation from Eq. 12 by knowing the initial weight matrix, *W* (plotted in Fig. 2 as prediction from theory based on *W*). Note that the prediction of this analysis by definition does not depend on *T*_p_, as it is based on stationary-state responses.

While the previous analysis sheds light on the relation between dynamics of the networks and the resulting weight changes via the weight matrix, it still needs to be evaluated numerically; in particular, calculating *A* = (*I* − *W*)^−1^ can be computationally expensive for large matrices and precludes further analytical insights into the key parameters underlying the emergence of different regimes of induction. We therefore developed a mean-field analysis to calculate average potentiation within the perturbed ensemble as a function of the average parameters of connectivity. The perturbed excitatory (E_1_), unperturbed excitatory (E_2_), and inhibitory (I) populations were reduced to single nodes in the mean-field analysis with the connectivity between them described by

$$\tilde{W}=(\begin{array}{ccc} w_{E_{1}\leftarrow E_{1}} & w_{E_{1}\leftarrow E_{2}} & w_{E_{1}\leftarrow I}\\ w_{E_{2}\leftarrow E_{1}} & w_{E_{2}\leftarrow E_{2}} & w_{E_{2}\leftarrow I}\\ w_{I\leftarrow E_{1}} & w_{I\leftarrow E_{2}} & w_{I\leftarrow I} \end{array})$$ (14)

For the connectivity matrix parameterized in Fig. 1G, and assuming that a fraction *f* of E neurons are perturbed (*f* = *N*_p_/*N*_E_), we can write the mean-field weight matrix as

$$\tilde{W}=(\begin{array}{ccc} f\;J_{\text{EE}} & \;(1-f)\;J_{\text{EE}} & -k\;J_{\text{EE}}\\ f\;J_{\text{EE}} & (1-f)\;J_{\text{EE}} & -k\;J_{\text{EE}}\\ \mathrm{kf}\;J_{\text{EE}} & k(1-f)\;J_{\text{EE}} & -k\;J_{\text{E}} \end{array})$$ (15)

where *J*_EE_ is the mean-field, overall coupling strength of E population. For a network with connection probability ϵ and average weight *J*_0_ of individual E➔E synapses, it can be expressed as *J*_EE_ = *N*_E_ϵ*J*_0_. The mean-field coupling of I➔{E,I} population can, in turn, be expressed as *N*_I_ϵ*gJ*_0_, where *g* determines the inhibition dominance of individual I➔{E,I} synapses over E➔E ones. If *N*_E_ = *N*_I_, and given similar connection probabilities for all connection types, *k* = *g*. Dominant individual E➔I synapses by the same factor also leads to overall dominance of E➔I coupling: *N*_E_ϵ*kJ*_0_, as expressed in mean-field couplings in Eq. 15.

Knowing the mean-field matrix $\tilde{W}$, we can now obtain the corresponding matrix of weight changes for the mean-field analysis, $\tilde{P}$, from Eq. 13, as

$$\tilde{P}=\eta \;\tilde{A}\delta \tilde{s}\delta {\tilde{s}}^{T}{\tilde{A}}^{T}$$ (16)

where $\delta \tilde{s}={\left(1\;0\;0\right)}^{T}$ and $\tilde{A}={\left(I-\tilde{W}\right)}^{-1}$. To obtain the average potentiation of synapses within the ensemble of perturbed neurons, we are interested in entry ${\tilde{p}}_{11}$ of the $\tilde{P}$ matrix, which can be obtained as

$${\tilde{P}}_{11}=\eta {\tilde{A}}_{11}^{2}$$ (17)

Writing *J* = *J*_EE_, ${\tilde{A}}_{11}$ can, in turn, be computed from $\tilde{A}={\left(I-\tilde{W}\right)}^{-1}$ as

$${\tilde{A}}_{11}=1-\frac{\mathrm{fJ}}{\Delta }(Jk^{2}-\mathrm{Jk}-1)$$ (18)

where Δ = *J*^2^*k*^2^ − *J*^2^*k* + *Jk* − *J* + 1. This is used in Fig. 2 for the mean-field analysis.

For *k* = 1, Eq. 18 suggests that ${\tilde{A}}_{11}\approx 1+\mathrm{fJ}$, and hence

$${\tilde{P}}_{11}\approx \eta \;{\left(1+\mathrm{fJ}\right)}^{2}$$ (19)

implying a supralinear enhancement of assembly formation for larger fraction of perturbed neurons. Note that this is the case independent of how large or small *J* is, especially whether *J* < 1 or *J* > 1 (unstable E-E subnetwork) does not change the result (cf. Fig. 2C). For large *J*, and strong *k* (*Jk* ≫ 1), another regime is obtained with ${\tilde{A}}_{11}\approx 1-f$, and

$${\tilde{P}}_{11}\approx \eta \;{\left(1-f\right)}^{2}$$ (20)

which suggests a weaker potentiation of synapses for larger *f* and hence *N*_p_ (cf. Fig. 2F).

## Notes

View/request a protocol for this paper from *Bio-protocol*.
