# Supplementary material for: Excitatory-inhibitory balance modulates the formation and dynamics of neuronal assemblies in cortical networks
Source: Sci Adv. 2021 Nov 3;7(45):eabg8411. doi: 10.1126/sciadv.abg8411 (PMC8565910; doi:10.1126/sciadv.abg8411)
Supplement: Supplementary file 1 — Figs. S1 to S7 [file sciadv.abg8411_sm.pdf]

Science Advances
AAAS

Supplementary Materials for

Excitatory-inhibitory balance modulates the formation and dynamics of neuronal assemblies in cortical networks

Sadra Sadeh* and Claudia Clopath*

*Corresponding author. Email: c.clopath@imperial.ac.uk (C.C.); s.sadeh@imperial.ac.uk (S.S.)

Published 3 November 2021, *Sci. Adv.* **7**, eabg8411 (2021)
DOI: 10.1126/sciadv.abg8411

This PDF file includes:

Figs. S1 to S7

Supplementary figures

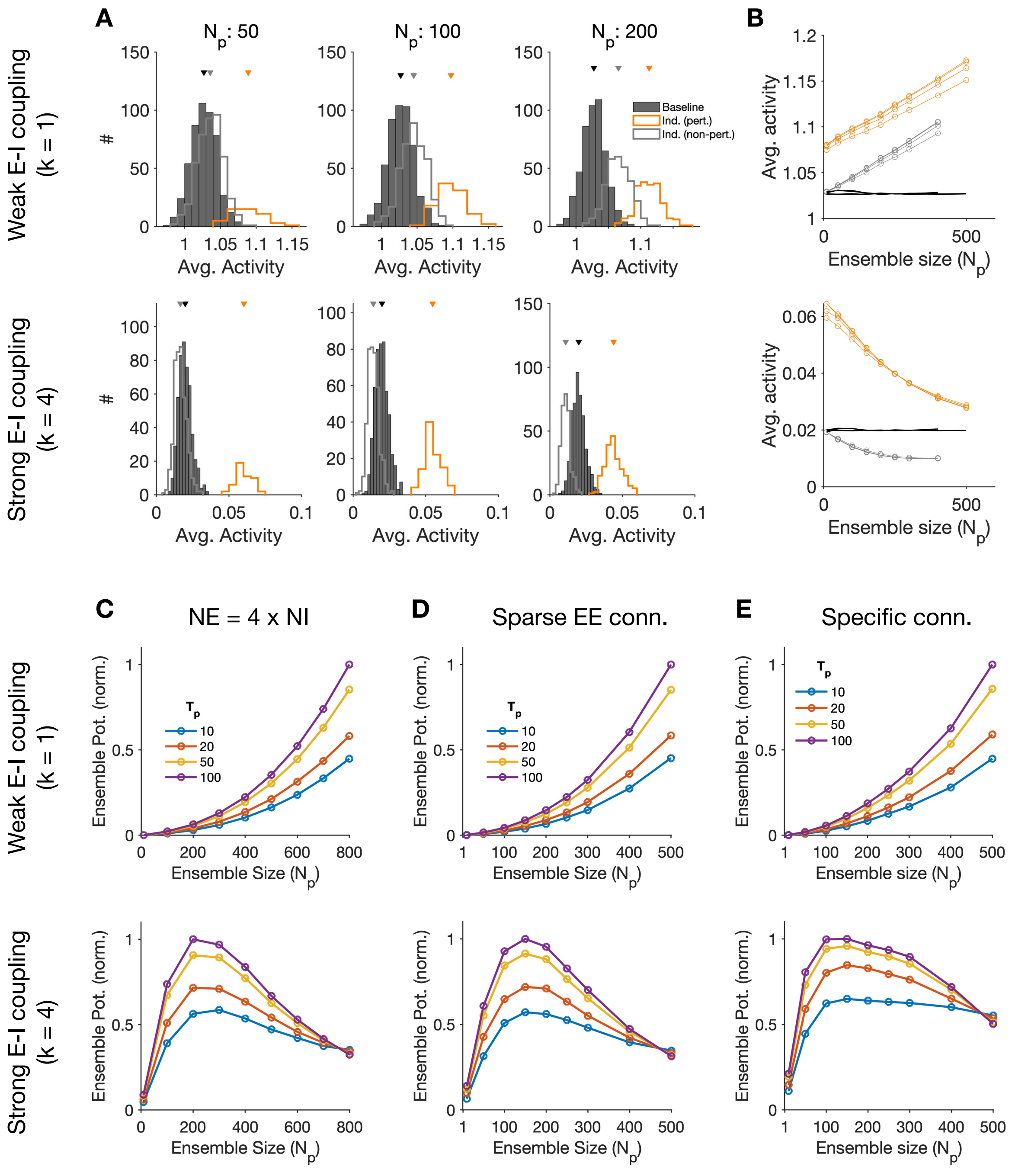

Fig. S1. Neuronal activity and induction of neuronal assemblies in different networks.
(A) Distribution of average activity of neurons in the networks shown in **Fig. 1** before induction (Baseline) and during induction (Ind.) for perturbed ensemble (pert.) and other excitatory neurons (non-pert.). Distributions are shown for perturbations sizes $N_p = 50$ (same as **Fig. 1G-I**), 100 and 200. **(B)** The average activity of population of perturbed (orange, pert.) and non-perturbed (grey, non-pert.) excitatory neurons during induction, compared to the average activity of the excitatory

population before induction (black, baseline) for different N_p. Different lines in each category denote different temporal patterns of perturbation ($T_p = 10, 20, 50, 100\ ms$). **(C)** Same as **Fig. 1J** for a network with larger number of excitatory neurons. $N_E = 800, N_I = 200$. To provide the same level of inhibition, I→{E,I} weights are made 4 times stronger to adjust for the lower number of inhibitory neurons. (**D**) Same as **Fig. 1J** for a network with sparser E-E connectivity. As opposed to all-to-all connectivity we assumed before, excitatory neurons are now connected to each other with 25% probability of connections ($\epsilon = 0.25$). To have the same level of overall E-E coupling, E→E weights are made 4 times stronger to adjust for the lower number of synapses. (**E**) Same as **Fig. 1J** for a network with specific recurrent connectivity. As opposed to network in **Fig. 1**, the weights of connections here are modulated by functional similarity of neurons, such that neurons with similarly assigned initial preferred features are connected more strongly together ($m = 0.5$; see Materials and methods for details, Eq. 3).

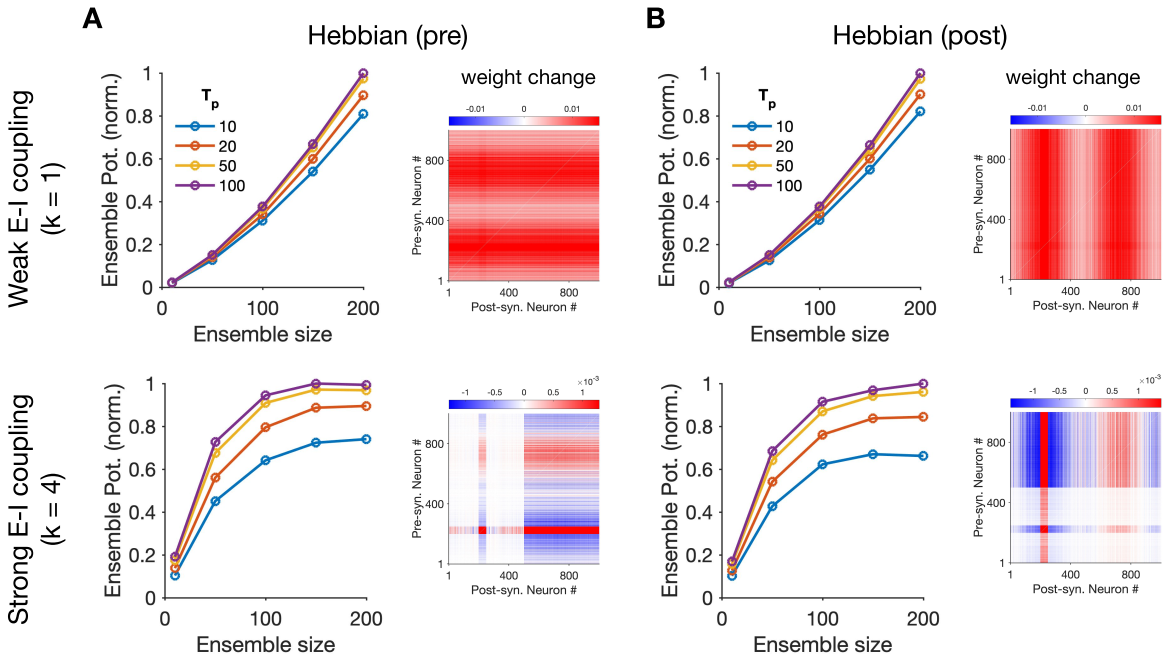

Fig. S2: Induction of neuronal assemblies in networks with different Hebbian rules.

The covariance-based Hebbian rule in **Fig. 1**, $\Delta w \propto (r_{pre} - r^0{}_{pre})(r_{post} - r^0{}_{post})$ (see Materials and methods), was governed by the deviation of pre- and post-synaptic activity from their baseline value before perturbations (denoted by r^0). This is changed here to consider different Hebbian-type rules of plasticity which depend only on the deviations of pre- (A) or post-synaptic (B) activity, while the absolute value of post- or pre-synaptic activity is preserved, respectively (see Materials and methods, Eq. 5). (**A**) Same as **Fig. 1J** when weight changes are governed by: $\Delta w \propto (r_{pre} - \mathrm{r}^0_{\mathrm{pre}})\, r_{post}$. (**B**) Same as **Fig. 1J** when weight changes are governed by: $\Delta w \propto \mathrm{r}_{\mathrm{pre}}(r_{post} - r^0{}_{post})$. The matrix of weight changes for $N_p = 50$ and $T_p = 50$ is shown for each condition on the right, respectively.

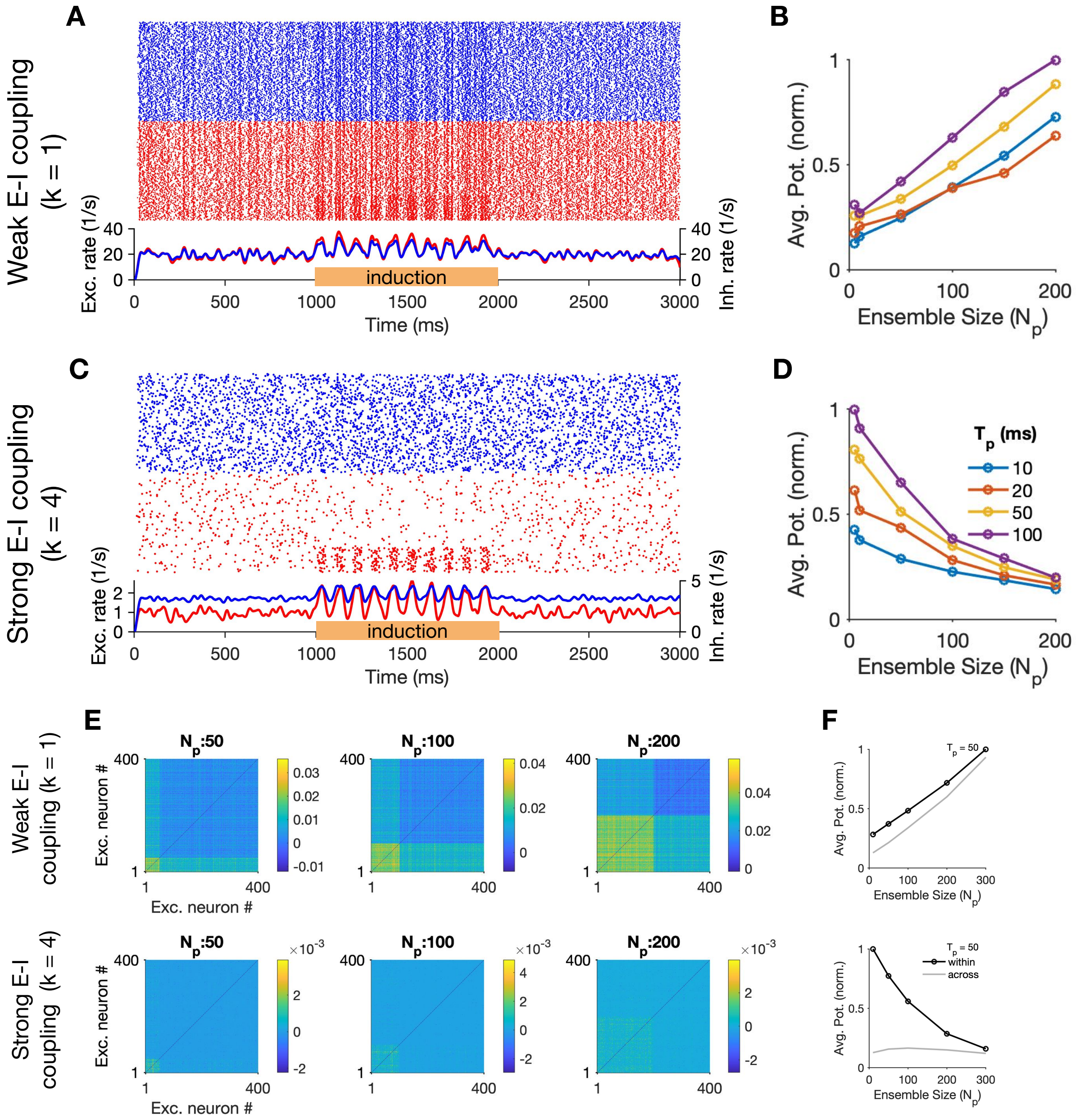

Fig. S3: **Induction of neuronal assemblies in spiking networks.**

(**A**) Induction of neuronal assemblies in networks of spiking neurons in the weak E-I coupling regime ($k = 1$). A sample simulation (with $N_p = 100$ and $T = 50\,ms$) is shown, with raster plots of activity on top (red: Exc., blue: Inh.), and average population activity of N_E Exc. and N_I Inh. neurons on the bottom (calculated in bins of 1 ms and smoothened with a sliding Gaussian kernel of 20 ms length). $N_E = N_I = 400$. Connectivity is all-to-all ($\epsilon = 1$) and random ($m = 0$), with an average weight of $w_{EE} = 0.1\,mV$ for E-to-E connections. $\tau^{\mathrm{E}}{}_m = \tau^{\mathrm{I}}{}_m = 20\,\mathrm{ms}$. Perturbations are performed for 10 cycles in this example, from 1000 ms to 2000 ms. (**B**) Average potentiation of individual synapses for induction protocols with different values of N_p and T_p shows similar dependence on the size of perturbed ensembles as rate-based models (cf. **Fig. 2A**). The

plasticity of synapses is governed by a Hebbian plasticity rule based on the covariances of pre- and post-synaptic sources. The pre- term is read from the presynaptic spiking activity of neurons and the post- term is inferred from the average free membrane potential of postsynaptic neurons (see Materials and methods). The absolute value of the average potentiation of synapses within the perturbed ensembles is normalized to the maximum value across all inductions. Perturbations are performed for 100 cycles to obtain more reliable estimates of response changes. (**C,D**) Same as (A,B) for induction in spiking networks with $k = 4$. (**E**) Implementation of a plasticity rule based on spike timing and voltage. Upon spiking activity in a pre-synaptic neuron j, the weight of connection to a post-synaptic neuron i is updated according to: $w_{ij} \leftarrow w_{ij} + dw_{ij}$, with $dw_{ij} = x_j v_i$. v_i is the free membrane potential of the post-synaptic neurons, and x_j is a trace of pre-synaptic activity: $\tau_p \mathrm{d}x_j/\mathrm{dt} = -x_j + \mathrm{a_p} \Sigma_n \delta(t - t_j^n)$. t_j^n is the time of the n-th spike of the j-th neuron. $\tau_p = 20\,ms$ and $a_p = 1$. Weight changes obtained from this rule is calculated for the baseline state before perturbations and for the activity during perturbations; weight changes in (E) are shown as the difference of the two. $T_p = 50\,ms$. (**F**) Average potentiation of weights in (E) within the perturbed ensemble (within) and across the perturbed ensemble and other neurons outside the ensemble (across) for different sizes of perturbed ensembles (N_p).

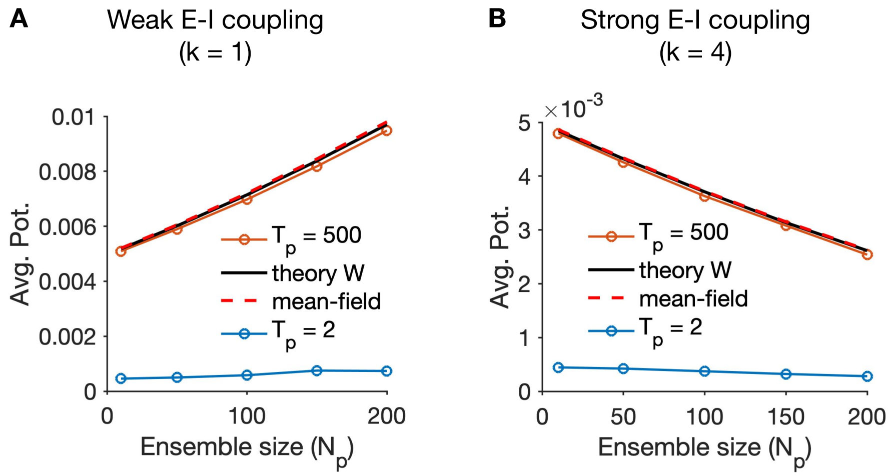

Fig. S4: Potentiation of synapses with extreme perturbation times.
(**A,B**) Same as **Fig. 2B,E**, respectively, for extremely small and large values of T_p. The residual discrepancy between theory and simulations (cf. **Fig. 2B,E**) is absent for very long pulses ($T_p = 500$), while the increasing/decreasing trends with the perturbed ensemble size are much weaker for very brief perturbations ($T_p = 2$).

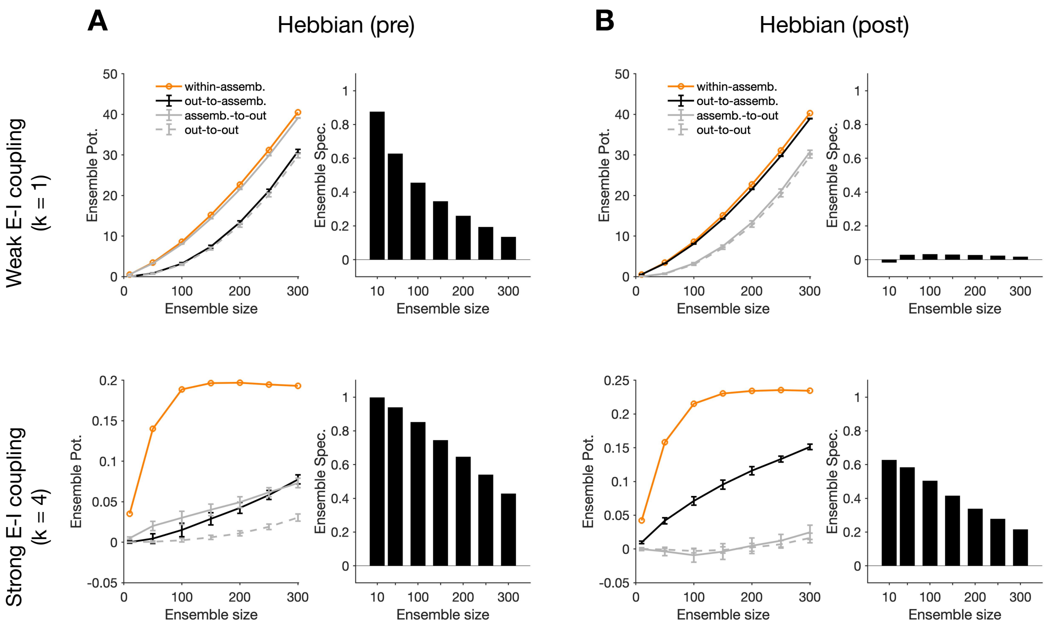

Fig. S5: Specificity of assembly formation with different Hebbian rules.

(**A,B**) Specificity of assembly formations (similar to **Fig. 3B-E**) for different Hebbian rules of plasticity. As opposed to the covariance-based rule in **Fig. 1** which depended on the response changes of both pre- and post-synaptic neurons (see Materials and methods), here the Hebbian rules depend on pre- (A) or post-synaptic (B) changes, while preserving the dependence on the absolute activity of the post and pre, respectively (similar to rules in **Fig. S2A,B**, respectively; see Materials and methods, Eq. 5, for details).

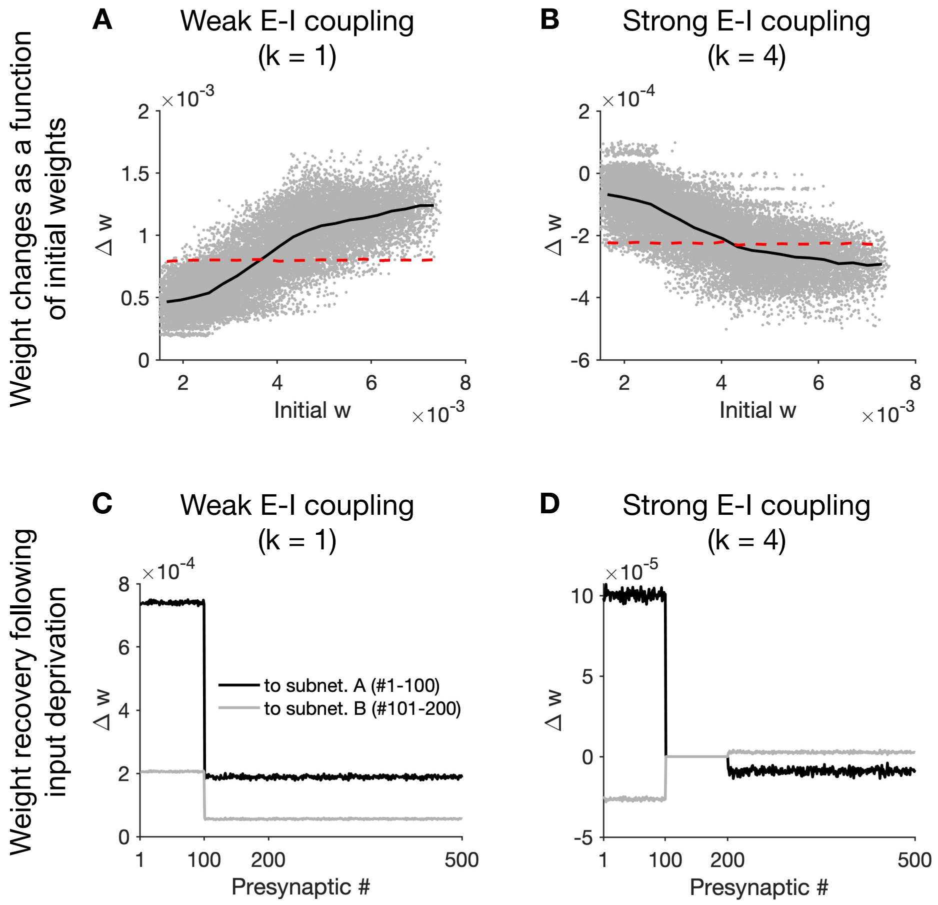

Fig. S6: Specificity of potentiation based on initial weights and after input deprivation. (**A,B**) Weight changes (Δw) of out-of-assembly connections (same as **Fig. S1E**, respectively), for different regimes of E-I coupling (A and B, respectively), as a function of their initial connectivity (Initial w) to the ensemble. The initial perturbed ensemble, namely the E neurons perturbed initially, are $N_p = 50$ neurons with the most similar preferred orientations (cf. **Fig. S1E**). The black lines show the average value of Δw calculated in 20 equal bins along the x-axis, respectively. The red lines show similar average values of Δw, when $N_p = 50$ neurons in the perturbed ensemble are chosen randomly, independent of their initial preferred orientations. (**C,D**) Weight changes of subnetworks with deprived input. In networks similar to those in **Fig. 2** (with random connectivity), the feedforward input to a fraction of neurons (#1-200) is reduced to half the initial value. Correlated input patterns (similar to those delivered in **Fig. 1**, with $T_p = 50$) from other sources are assumed to activate one of the subnetworks (A: #1-100), while the other subnetwork (B: #101-200) does not receive the input perturbations (Materials and methods). The average weight changes of presynaptic E neurons ($N_E = 500$) to different subnetworks (cf. e.g. **Fig. 2**) are plotted, for weak (C) and strong (D) E-I coupling regimes, respectively.

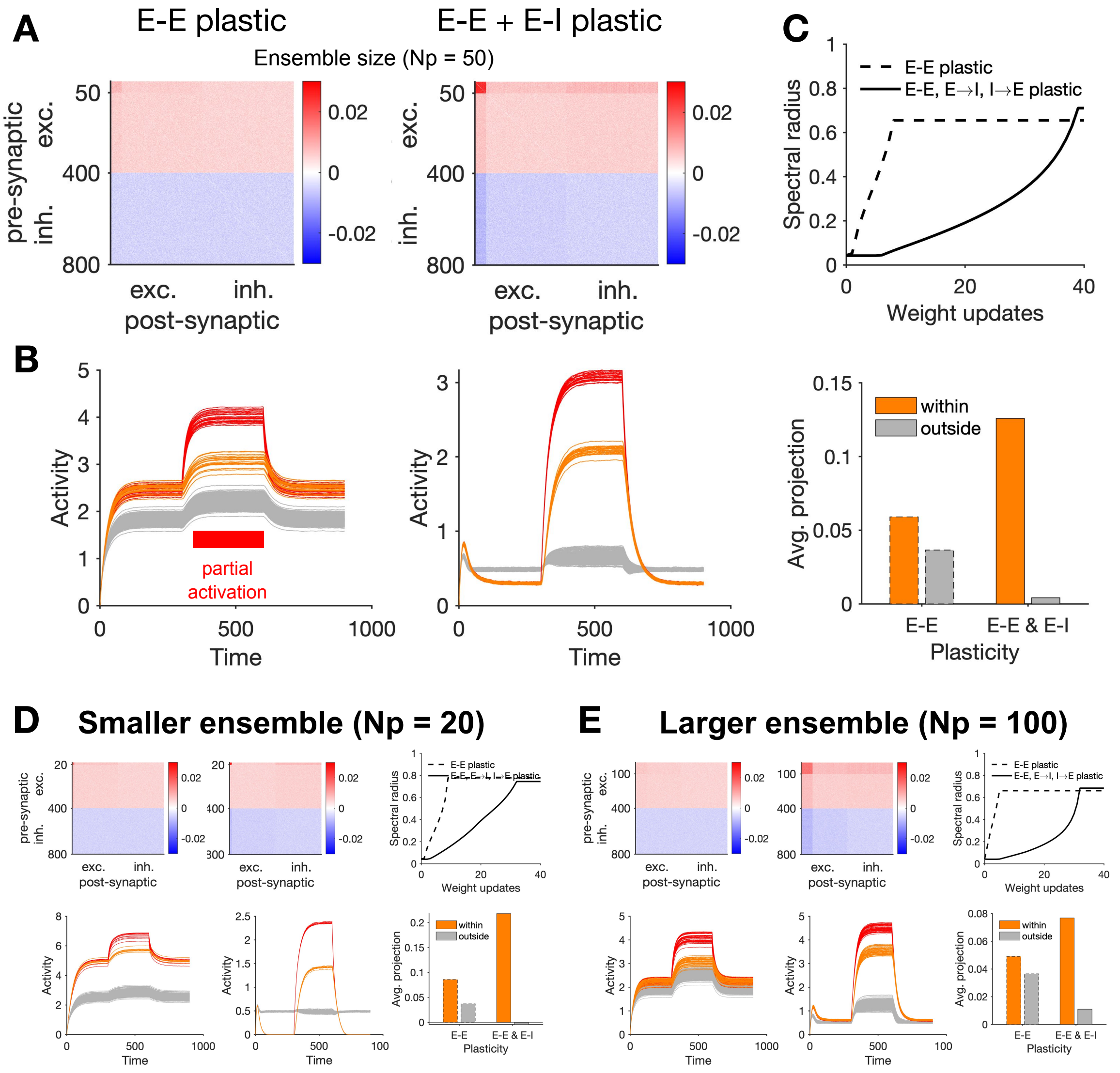

Fig. S7: Transition between regimes of induction with different rules of E-I learning. (**A-C**) Same as **Fig. 7** for a different rule of E-I plasticity. E→I and I→E plasticity in **Fig. 7** depended on changes in the activity of pre-synaptic excitatory and inhibitory neurons, respectively (Materials and methods). Here, pre- and post-synaptic activity changes are both considered for both types of synapses. $N_p = 50$, $\eta_{EE} = 0.05$, $\eta_{EI} = \eta_{IE} = 0.1$. (**D,E**) Same as (A-C), respectively, for different sizes of perturbed ensembles: (D) $N_p = 20$, $\eta_{EE} = 0.2$, $\eta_{EI} = \eta_{IE} = 0.4$; (E) $N_p = 100$, $\eta_{EE} = 0.025$, $\eta_{EI} = \eta_{IE} = 0.05$.
